# Multi-Pesticide Residue Occurrence and Age-Specific Dietary Exposure Risk Assessment of Apples from Major Production Areas in Xinjiang, China

**DOI:** 10.3390/foods15193491

**Published:** 2026-09-29

**Authors:** Yuqi Mo, Jiuyang Zhao, Xiangtian Wang, Caiqin Xu, Shaoli Fan, Jianjun Yang, Lu Yang

**Affiliations:** 1 College of Ecology and Environment, Xinjiang University, Urumqi 830017, China; uiiio697@163.com (Y.M.);; 2 Key Laboratory of State Forestry and Grassland Administration on Desert Oasis Ecosystem Protection and Restoration, Xinjiang Key Laboratory of Fruit Tree Species Breeding and Cultivation, Xinjiang Academy of Forestry, Urumqi 830000, China

**Keywords:** Xinjiang apples, pesticide residues, age-specific dietary exposure, cumulative exposure, multi-pesticide co-occurrence, risk contribution

## Abstract

To characterize pesticide residues and age-specific dietary exposure risks in apples from Xinjiang’s main production areas, 120 samples were collected from Aksu, Ili, and Kashgar in October 2025 and screened for 82 pesticides using GC–MS/MS, LC–MS/MS, and GC–ECD. Chronic, acute, and cumulative dietary exposure was subsequently assessed. Eight pesticides were detected, all below their corresponding maximum residue limits (MRLs). Acetamiprid had the highest detection frequency (28.3%), yet pyridaben was the leading contributor to chronic hazard index (HI_c_) under both lower-bound (LB) and upper-bound (UB) scenarios. Carbendazim, acetamiprid, and pyridaben were the major contributors to summed acute hazard index (HI_a_), demonstrating a mismatch between detection frequency and risk contribution. The sample-level chronic hazard index was significantly higher in the multi-pesticide co-occurrence group than in the single-residue group (*p* < 0.001). Toddlers showed the highest cumulative exposure, with chronic hazard indices of 0.179% (LB) and 2.209% (UB), and an acute hazard index of 33.759%. Sensitivity analysis revealed that adopting the recently updated EFSA toxicological reference values for acetamiprid caused acute hazard quotients to exceed the 100% screening threshold in toddlers and children, highlighting how the source and currency of toxicological reference values can affect risk assessment conclusions. Under the primary analysis reference values, dietary exposure risks were low; however, risk prioritization should account not only for detection frequency and MRL compliance but also for actual exposure levels, co-occurrence patterns, and updates to toxicological reference values.

## 1. Introduction

Pesticide residues in fruits and the resulting dietary exposure are central concerns in food safety assessment [1]. China is the world’s largest producer and consumer of apples, leading globally in both cultivation area and output. In 2025, China’s total apple production reached 51.5555 million tonnes, accounting for more than 50% of global output [2]. Xinjiang is a key specialty apple-producing region in China, and its abundant solar radiation and large diurnal temperature variation have shaped major production areas in Aksu, Ili, and Kashgar. Xinjiang’s apple production reached 2.4535 million tonnes in 2025, a year-on-year increase of 6.9% [3]. The scale of Xinjiang’s apple industry and its expanding market reach underscore the need for pesticide residue monitoring and age-stratified dietary exposure risk assessment. Apples, however, have a relatively long growing season and are susceptible to a range of pests and diseases; insecticides, acaricides, fungicides, and other pesticides are therefore commonly applied for crop protection, and the residue level remaining in fruit at harvest is influenced by pesticide-specific dissipation behavior, application timing, and the pre-harvest interval [4]. Food safety oversight currently relies primarily on maximum residue limits (MRLs) to determine sample compliance, but MRL compliance does not equate to dietary exposure safety—exposure levels are also shaped by residue concentration, food consumption, body weight, and toxicological reference values such as the acceptable daily intake (ADI) and acute reference dose (ARfD). Relying on MRL compliance alone is therefore insufficient to capture the actual exposure risk faced by different population groups [5,6].

In dietary exposure assessment, the hazard quotient (HQ) and hazard index (HI) are widely used quantitative tools that integrate residue concentrations, dietary consumption parameters, and toxicological reference values to evaluate exposure to single and multiple pesticides, respectively [7]. Previous studies have shown that detection frequency, residue concentration, and risk contribution do not always follow the same ranking: a pesticide with a high detection frequency does not necessarily contribute the most to risk [8], whereas a pesticide with a relatively low toxicological reference value may still account for a substantial share of cumulative risk even when detected less frequently [9]. Detection frequency or MRL compliance alone therefore cannot reliably identify the pesticides that contribute most to risk, and current regulatory frameworks lack a tiered classification based on cumulative exposure levels.

As one of China’s major apple-producing regions, Xinjiang—particularly its Aksu, Ili, and Kashgar production areas—remains insufficiently characterized in terms of pesticide residue patterns and associated dietary exposure risks. Moreover, while multi-pesticide residues may occur within the same sample, it remains unclear whether such co-occurrence is associated with higher sample-level cumulative exposure than single-residue occurrence [10]. Another unresolved issue is whether changes in toxicological reference values can substantially alter risk estimates and pesticide prioritization [11]. To address these gaps, the present study goes beyond conventional residue detection and MRL-based evaluation by integrating regional residue profiling, age-specific dietary exposure assessment, sample-level multi-pesticide co-occurrence analysis, and sensitivity analysis of toxicological reference values.

Against this background, 120 apple samples were collected from three major apple-producing regions of Xinjiang—Aksu, Ili, and Kashgar—and screened for 82 pesticides. Residue occurrence and regional variation were characterized, and chronic, acute, and screening-level cumulative dietary exposure was assessed for five age groups using age-specific food consumption and body-weight parameters. Detection frequency and risk-contribution rankings were compared, and the cumulative exposure characteristics of multi-pesticide co-occurrence samples were further evaluated. A sensitivity analysis was conducted to assess the effect of updated toxicological reference values on the risk assessment results, with the aim of providing a basis for pesticide residue risk identification and differentiated monitoring in Xinjiang apples.

## 2. Materials and Methods

### 2.1. Sample Collection and Preparation

**Figure 1:**
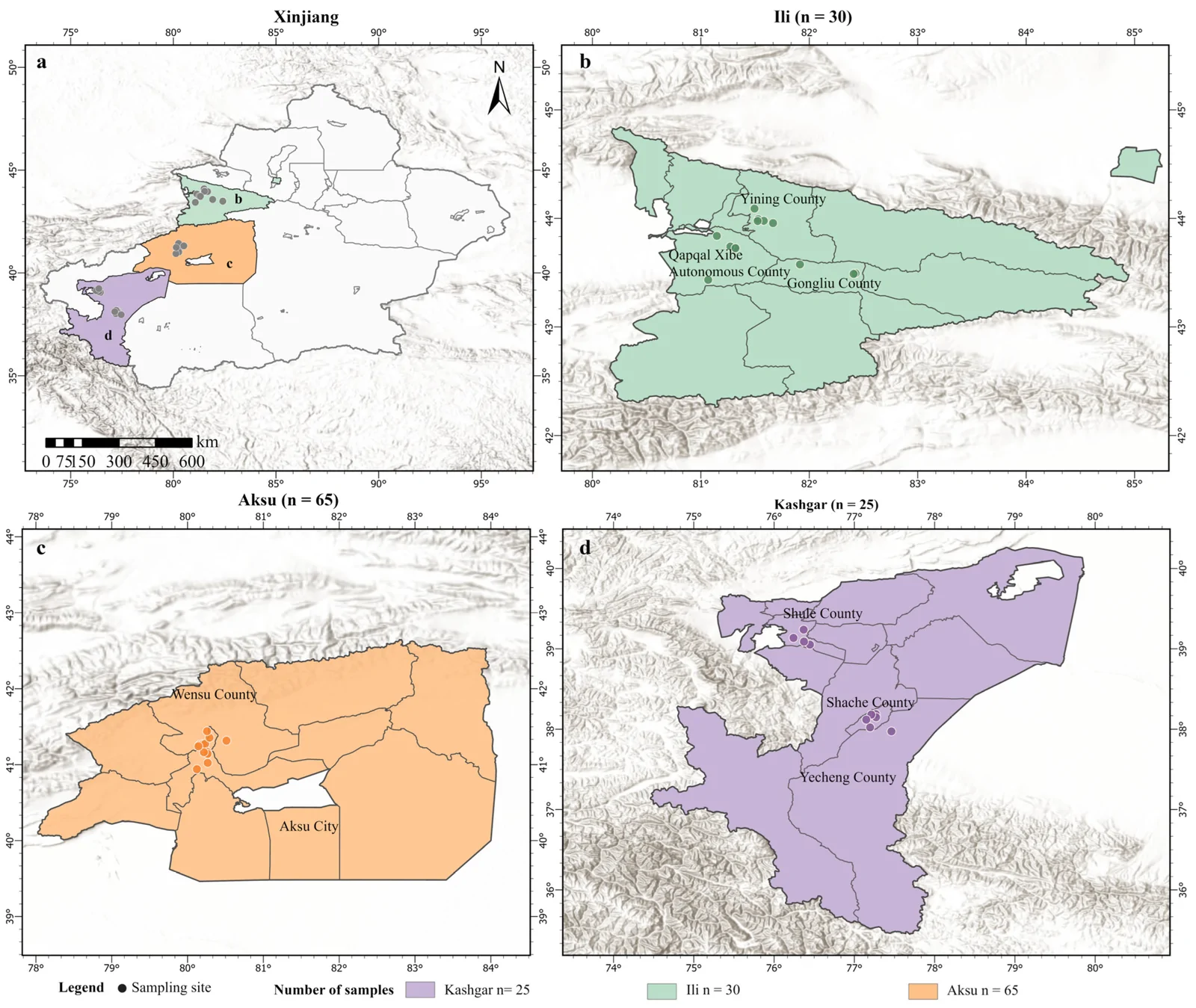
Apple sampling locations in Xinjiang, China. (**a**) locations of the three study regions within Xinjiang; (**b**) sampling sites in Ili (*n* = 30); (**c**) sampling sites in Aksu (*n* = 65); and (**d**) sampling sites in Kashgar (*n* = 25).

In October 2025, a total of 120 apple samples were collected from nine counties across three major apple-producing regions of Xinjiang, China: Aksu Prefecture (*n* = 65), Ili Kazak Autonomous Prefecture (*n* = 30), and Kashgar Prefecture (*n* = 25) (Figure 1). At each sampling site, a representative orchard was selected, and 8–10 apple trees were chosen from both the central and peripheral areas of the orchard. Apples were randomly picked from the upper, middle, and lower canopy, at both inner and outer positions, and pooled to form one composite sample per site. Samples were packed in clean sample bags and transported to the laboratory within 1–3 days under cold-chain conditions. Upon arrival, whole apples were homogenized without washing or removing the stems and cores, subdivided into aliquots, and stored at −20 °C until analysis.

### 2.2. Instruments and Reagents

GC–MS/MS and LC–MS/MS analyses were performed using a Xevo TQ-GC system and a Xevo TQ system, respectively (both from Waters Corporation, Milford, MA, USA). Chlorothalonil was determined using an Agilent 8890 gas chromatograph (Agilent Technologies, Santa Clara, CA, USA) equipped with an electron capture detector (ECD). Other equipment included a Multifuge X1R refrigerated centrifuge (Thermo Fisher Scientific, Waltham, MA, USA) and an HSC-17-193 nitrogen evaporator (Shanghai Hongji Instrument Co., Ltd., Shanghai, China).

Pesticide reference standards (1000 μg/mL stock solutions) were purchased from Anpel-Trace Standard Technical Services (Shanghai) Co., Ltd. (Shanghai, China). Working solutions were prepared by serial dilution to a 10 μg/mL intermediate standard, using acetonitrile for LC–MS/MS and ethyl acetate for GC–MS/MS, then further diluted with blank matrix extract to obtain matrix-matched calibration solutions. Heptachlor epoxide-B (purity, 97.0%; product No. CDAA-411017-10mg) was obtained from Anpel-Trace Standard Technical Services (Shanghai) Co., Ltd. (Shanghai, China) and was used as the internal standard for GC–MS/MS analysis. A 5 μg/mL internal-standard working solution was prepared in ethyl acetate.

### 2.3. Pesticide Residue Determination and Quality Control

#### 2.3.1. Sample Preparation

Pesticide residues in apple samples were determined according to GB 23200.113-2018 (National Food Safety Standard—Determination of 208 Pesticides and Their Metabolites in Foods of Plant Origin by Gas Chromatography–Tandem Mass Spectrometry) [12] and GB 23200.121-2021 (National Food Safety Standard—Determination of 331 Pesticides and Their Metabolites in Foods of Plant Origin by Liquid Chromatography–Tandem Mass Spectrometry) [13],and by GC–ECD for chlorothalonil according to NY/T 761-2008 [14]. The analytical panel covered 82 pesticides: 70 by GC–MS/MS, 11 by LC–MS/MS, and chlorothalonil by GC–ECD. Residue results were evaluated against the maximum residue limits (MRLs) specified in GB 2763-2021 (National Food Safety Standard—Maximum Residue Limits for Pesticides in Food) [15].

A 10.0 g portion of the homogenized sample was accurately weighed into a 50 mL centrifuge tube, followed by the addition of 10 mL of acetonitrile and a ceramic homogenizer. The mixture was shaken for 1 min, after which the QuEChERS extraction salts (Shanghai ANPEL Laboratory Technologies Inc., Shanghai, China, SBEQ-CA1555) (containing 4 g of anhydrous MgSO_4_, 1 g of NaCl, 1 g of sodium citrate, and 0.5 g of disodium hydrogen citrate sesquihydrate) were added. After further shaking for 1 min, the mixture was centrifuged at 4200 rpm for 5 min. A 6 mL aliquot of the supernatant was transferred to a QuEChERS cleanup tube (containing 900 mg of anhydrous MgSO_4_ and 150 mg of PSA), vortex-mixed for 1 min, and centrifuged at 4200 rpm for 5 min.

For LC–MS/MS analysis, the purified extract was passed through a membrane filter and injected directly into the instrument. For GC–MS/MS analysis, a 2 mL aliquot of the purified extract was evaporated to near dryness under a stream of nitrogen in a 40 °C water bath, after which 20 μL of internal standard solution was added and the residue was reconstituted in 1 mL of ethyl acetate before injection.

For chlorothalonil analysis, a 25.0 g portion of homogenized apple sample was extracted with 50 mL of acetonitrile by high-speed homogenization for 2 min. The extract was filtered into a stoppered graduated cylinder containing 5–7 g of sodium chloride, shaken vigorously for 1 min, and allowed to stand for 30 min for phase separation. A 10 mL aliquot of the acetonitrile phase was evaporated to near dryness, reconstituted with acetone, and brought to a final volume of 5.0 mL before GC–ECD analysis.

#### 2.3.2. Instrumental Conditions

Eleven pesticides were analyzed by LC–MS/MS according to GB 23200.121-2021. Chromatographic separation was performed on an ACQUITY UPLC BEH C18 column (2.1 mm × 100 mm, 1.7 μm). The mobile phase consisted of 2 mmol/L ammonium formate–formic acid in water (A) and 2 mmol/L ammonium formate–formic acid in methanol (B). The gradient was as follows: 0–1 min, 97% A; 1.5 min, 85% A; 2.5 min, 50% A; 18 min, 30% A; 23–27 min, 2% A; and 27.1–30 min, 97% A. The column temperature was maintained at 40 °C, and the injection volume was 2 μL. Electrospray ionization (ESI) in positive-ion mode with multiple reaction monitoring (MRM) was used.

Seventy pesticides were analyzed by GC–MS/MS according to GB 23200.113-2018 using a VF-1701ms capillary column (30 m × 0.25 mm × 0.25 μm). The oven temperature was initially held at 40 °C for 1 min, increased to 120 °C at 40 °C/min, then to 240 °C at 5 °C/min, and finally to 300 °C at 12 °C/min, followed by a 6 min hold. Helium (purity 99.999%) was used as the carrier gas at a flow rate of 1.0 mL/min. The injector temperature was 280 °C, and 1 μL was injected in pulsed splitless mode. Electron ionization (EI, 70 eV) was used, with an ion source temperature of 250 °C and a transfer line temperature of 280 °C. Data were acquired in MRM mode using heptachlor epoxide-B as the internal standard.

Chlorothalonil was determined by GC–ECD according to NY/T 761-2008 [14] using an HP-5 capillary column (30 m × 0.32 mm × 0.25 μm). The oven temperature was initially held at 150 °C for 2 min, increased to 270 °C at 6 °C/min, and held for 8 min. Nitrogen (purity 99.999%) was used as the carrier gas at a flow rate of 1.5 mL/min. The injector temperature was 200 °C, the injection volume was 1 μL, and injection was performed in splitless mode. An electron capture detector was operated at 320 °C.

Compound-specific quantitative and qualitative MRM transitions and the corresponding collision energies for the LC–MS/MS and GC–MS/MS analytes are provided in the Supplementary Table S3.

#### 2.3.3. Calibration and Method Validation

Quantification was performed using analyte-specific calibration curves. For LC–MS/MS and GC–MS/MS analyses, matrix-matched calibration standards were prepared using blank apple extracts processed in the same manner as the test samples. The calibration range was 0.005–1 mg/L for the LC–MS/MS analytes. For GC–MS/MS, 52 pesticides were calibrated over 0.005–1 mg/L and 18 over 0.01–2 mg/L. Chlorothalonil was calibrated over 0.005–0.6 mg/L. Individual calibration ranges are provided in the Supplementary Table S3.

For LC–MS/MS, calibration curves were constructed by plotting analyte peak area against concentration. For GC–MS/MS, the internal standard method was used, with heptachlor epoxide-B as the internal standard and the analyte/internal-standard peak-area ratio plotted against the corresponding concentration ratio. Chlorothalonil was quantified by external calibration. All calibration curves showed R^2^ values greater than 0.99.

The LOD for each pesticide was determined from 12 independently prepared low-concentration samples at 0.005 mg/kg and calculated as 3 × SD. Experimentally determined LODs and the individual LOQs are provided in the Supplementary Table S3. LOQs were based on the corresponding standard methods. The eight pesticides detected in the apple samples all had an LOQ of 0.01 mg/kg.

Analytical results were classified into three categories: non-detects, detected below the LOQ (<LOQ), and quantified results. Results that failed to meet the qualitative identification criteria of the corresponding analytical method were classified as non-detects, whereas those meeting the criteria were considered detected. Among the detected results, those with estimated concentrations below the LOQ were reported as <LOQ, while those at or above the LOQ were considered quantified. Results reported as <LOQ were included in the occurrence and co-occurrence analyses but were excluded from calculations of mean residue concentrations and comparisons with MRLs.

Spiked-recovery experiments were conducted at a fortification level of 0.1 mg/kg for each pesticide. For the LC–MS/MS and GC–MS/MS analyses, three separate 10.0 g portions of homogenized blank apple were independently fortified before extraction with 100 μL of the corresponding working standard solution, in which each target pesticide was present at 10 μg/mL. For chlorothalonil analyzed by GC–ECD, three separate 25.0 g portions of homogenized blank apple were fortified with 250 μL of a 10 μg/mL chlorothalonil working standard solution. The fortified samples were then processed according to the corresponding sample-preparation and instrumental procedures used for the study samples. Mean recoveries for the 82 pesticides ranged from 81.5% to 119.4%, with relative standard deviations (RSDs) ranging from 0.09% to 15.52%.

#### 2.3.4. Quality Assurance and Quality Control

Quality assurance and quality control procedures were applied throughout the analysis. One real sample from every 10 samples was independently prepared and analyzed in triplicate to assess analytical repeatability. Procedural blanks and blank apple matrix samples were included in the analytical batches to monitor potential contamination and matrix background. Fortified matrix QC samples were analyzed at a frequency of one per 10 real samples, with acceptable recoveries set at 80–120%.

### 2.4. Dietary Exposure Risk Assessment

Dietary exposure risk assessment was conducted using pesticide residue concentrations in apple samples and age-specific apple consumption data [16]. The study population was divided into five age groups: toddlers, children, adolescents, adults, and older adults. The mean body weight, mean daily apple consumption, large-portion consumption, and mean unit weight of an apple for each age group are presented in Table 1. Chronic and acute dietary exposure risks were characterized using the chronic hazard quotient (HQ_c_) and acute hazard quotient (HQ_a_), respectively.

For all results below the limit of quantification (LOQ), including nondetects and instrument signals corresponding to concentrations below the LOQ, lower-bound (LB) and upper-bound (UB) scenarios were applied in accordance with the principles for handling left-censored data described by the European Food Safety Authority (EFSA). Under the LB scenario, values below the LOQ were assigned a value of zero, whereas under the UB scenario, they were assigned the method LOQ of 0.01 mg/kg [18]. Based on these assignments, the mean residue concentration of each pesticide across all 120 apple samples was calculated separately under the LB and UB scenarios and denoted as C_LB_ and C_UB_, respectively.

Chronic dietary exposure from apple consumption was estimated using a deterministic approach that combines mean residue concentrations with age-specific mean consumption and body weight [19]. The estimated daily intake was calculated separately under the LB and UB scenarios as follows:

$$EDI_{LB,i,g}=\frac{C_{LB,i}\times F_{g}}{bw_{g}}$$

$$EDI_{UB,i,g}=\frac{C_{UB,i}\times F_{g}}{bw_{g}}$$

where *EDI*_LB,*i,g*_ and *EDI*_UB,*i,g*_ are the estimated daily intakes of pesticide *i* for age group *g* under the LB and UB scenarios, respectively (mg/kg bw/day); *C*_LB,*i*_ and *C*_UB,*i*_ are the mean residue concentrations of pesticide *i* across all 120 samples under the corresponding scenarios (mg/kg); *F_g_* is the mean daily apple consumption of age group *g* (kg/day); and bw_g_ is the mean body weight of that age group (kg).

The chronic hazard quotients under the two scenarios were calculated as:

$$HQ_{c,LB,i,g}=\frac{EDI_{LB,i,g}}{ADI_{i}}\times 100\%$$

$$HQ_{c,UB,i,g}=\frac{EDI_{UB,i,g}}{ADI_{i}}\times 100\%$$

where *ADI_i_* is the acceptable daily intake of pesticide *i* (mg/kg bw/day). An *HQ_c_*_,LB_ or HQ_c,UB_ value below 100% indicates that the estimated chronic exposure under the corresponding scenario is below the adopted *ADI*.

Acute dietary exposure was estimated using an equation based on Case 2a of the International Estimated Short-Term Intake (IESTI) approach, with the highest observed residue concentration for each pesticide used as the residue input [20]. The acute hazard quotient (HQ_a_) was then calculated as follows:

$$HQ_{a,i,g}=\frac{U\times HR_{i}\times v+\left(LP_{g}-U\right)\times HR_{i}}{bw_{g}\times ARfD_{i}}\times 100\%$$

where *U* is the mean unit weight of an apple (0.255 kg); *HR_i_* is the highest residue concentration of pesticide *i* (mg/kg); v is the variability factor, set at 3; *LP_g_* is the large-portion apple consumption of age group *g* (kg/day); *bw_g_* is the mean body weight of that age group (kg); and *ARfD_i_* is the acute reference dose of pesticide *i* (mg/kg bw).

Acute exposure was not divided into LB and UB scenarios. Among the six pesticides included in the acute risk assessment, the HR values of five pesticides were based on confirmed quantitative results at or above the method LOQ. For imidacloprid, the highest instrument signal corresponded to a concentration of 0.006 mg/kg, which was below the method LOQ of 0.01 mg/kg but within the calibration range, whose lowest calibration point was 0.005 mg/kg. Because acute exposure assessment is intended to characterize high-end exposure associated with a single large consumption event, this value was used as a provisional screening-level HR for imidacloprid. As this concentration was below the method LOQ and was not independently validated at that level, the resulting HQ_a_ was interpreted as a screening-level estimate. Although spirodiclofen also produced an instrument signal corresponding to 0.006 mg/kg, it was not included in the acute risk calculation because no numerical ARfD was available. Etoxazole was excluded for the same reason.

An HQ_a_ value below 100% indicates that the estimated acute exposure is below the adopted ARfD.

The cumulative chronic hazard indices under the LB and UB scenarios were calculated separately as

$$HI_{c,LB,g}=\sum_{i=1}^{8}HQ_{c,LB,i,g}$$

$$HI_{c,UB,g}=\sum_{i=1}^{8}HQ_{c,UB,i,g}$$

The cumulative acute hazard index was calculated as

$$HI_{a,g}=\sum_{i=1}^{6}HQ_{a,i,g}$$

where *HI_c,_*_LB,*g*_ and *HI_c,_*_UB,*g*_ are the cumulative chronic hazard indices for age group *g* under the LB and UB scenarios, respectively, and *HI_a,g_* is the cumulative acute hazard index. All eight pesticides were included in the chronic cumulative assessment, whereas only the six pesticides with numerical ARfD values were included in the acute cumulative assessment. Because the highest residue (HR) values for each pesticide were selected independently and may originate from different apple samples, the summed acute hazard index (HI_a_) represents a conservative worst-case screening scenario rather than the acute cumulative exposure associated with any single sample or consumption event.

An *HI_c_* or *HI_a_* value below 100% indicates that the screening-level cumulative exposure does not exceed the corresponding threshold. Because the detected pesticides may act through different toxicological modes of action, the summed hazard index was used only as a conservative screening metric to characterize the overall relative exposure burden and to identify samples or pesticides warranting further attention [21]. It was not intended to represent a mechanism-based cumulative risk assessment.

To examine the relationship between multi-pesticide co-occurrence and cumulative exposure, sample-level chronic hazard quotients were calculated for the 50 samples classified as positive in the pesticide-occurrence analysis. Samples containing one pesticide were assigned to the single-residue group, whereas those containing two or more pesticides were assigned to the multi-pesticide co-occurrence group.

For each sample, results below the LOQ and nondetects were assigned values according to the LB and UB rules described above. The sample-level chronic cumulative hazard indices were then calculated as

$$HI_{c,sample,LB,j}=\sum_{i=1}^{8}HQ_{c,sample,LB,i,j}$$

$$HI_{c,sample,UB,j}=\sum_{i=1}^{8}HQ_{c,sample,UB,i,j}$$

where *j* represents an individual apple sample. Because toddlers had the highest ratio of mean apple consumption to body weight, the mean consumption and body-weight parameters for toddlers were used in the sample-level comparison and correlation analysis.

The sample-level indices were used to compare relative cumulative exposure among samples with different pesticide-residue patterns. They were not interpreted as evidence of synergistic, antagonistic, or other confirmed combined toxic effects [22,23].

The contribution of each pesticide to cumulative chronic risk was calculated separately under the LB and UB scenarios:

$$RC_{c,LB,i,g}=\frac{HQ_{c,LB,i,g}}{HI_{c,LB,g}}\times 100\%$$

$$RC_{c,UB,i,g}=\frac{HQ_{c,UB,i,g}}{HI_{c,UB,g}}\times 100\%$$

The contribution of each pesticide to cumulative acute risk was calculated as

$$RC_{a,i,g}=\frac{HQ_{a,i,g}}{HI_{a,g}}\times 100\%$$

where *RC_c_*_,LB,*i,g*_ and *RC_c,_*_UB*,i,g*_ represent the contribution of pesticide *i* to cumulative chronic risk for age group *g* under the LB and UB scenarios, respectively, and *RC_a,i,g_* represents its contribution to cumulative acute risk. Pesticides were ranked according to their contribution rates to identify the major contributors to cumulative dietary exposure.

The ADI and ARfD values adopted for the eight pesticides and their sources are presented in Table 2. For pesticides other than acetamiprid, EFSA values were adopted because no applicable JMPR assessment was available or the JMPR and EFSA values were consistent. For acetamiprid, the primary analysis used the JMPR reference values, with an ADI of 0.07 mg/kg bw/day and an ARfD of 0.10 mg/kg bw [24]. The selected ADI was also consistent with the value specified in GB 2763-2021.

To evaluate the influence of toxicological reference value selection, a sensitivity analysis was conducted for acetamiprid using the EFSA 2024 reference values, with an ADI of 0.005 mg/kg bw/day and an ARfD of 0.005 mg/kg bw. Residue concentrations, apple consumption, body weight, and all other exposure parameters were held constant, and the chronic and acute hazard quotients were recalculated for each age group [25]. The results of the sensitivity analysis are presented in Section 3.7.

To complement the primary 2025 dietary exposure assessment, a supplementary analysis was performed using the 2024 regional monitoring data. This analysis included 12 pesticides detected in 2024 but not included in the 2025 analytical panel. The assessment focused on toddlers, who had the highest estimated exposure based on the consumption and body-weight parameters used in this study. Chronic exposure was estimated under lower-bound (LB) and upper-bound (UB) scenarios using the same equations, consumption parameters, and procedures for handling results below the LOQ as described above. Acute exposure was estimated using the highest residue concentration observed in the 2024 dataset for each pesticide with an applicable numerical ARfD. The toxicological reference values used and the supplementary assessment results are presented in Table S4.

### 2.5. Statistical Analysis

Data compilation, formatting, and preliminary statistical analyses were performed using Microsoft Excel. Pesticide residue occurrence, residue concentrations, and dietary exposure risk parameters were analyzed using descriptive statistics, including the number of samples, detection rate, mean, median, interquartile range (IQR), and extreme values. Differences in detection rates among production regions were compared using Pearson’s chi-squared test. Under both the lower-bound (LB) and upper-bound (UB) scenarios, the Mann–Whitney U test was used to compare the sample-level chronic cumulative risk index (HI_c,sample_) between the single-residue group and the multi-residue co-occurrence group, and Cliff’s delta (δ) [26] was calculated to evaluate the effect size of the between-group difference. The relationship between the number of detected pesticides and HI_c,sample_ was assessed using Spearman’s rank correlation analysis. All statistical tests were two-sided, with a *p*-value < 0.05 considered statistically significant. The sampling site distribution map was generated using ArcGIS Pro3.6.0; all other statistical analyses and figures were primarily produced using Python 3.13.14.

## 3. Results

### 3.1. Overall Characteristics of Pesticide Residues in Apples

**Figure 2:**
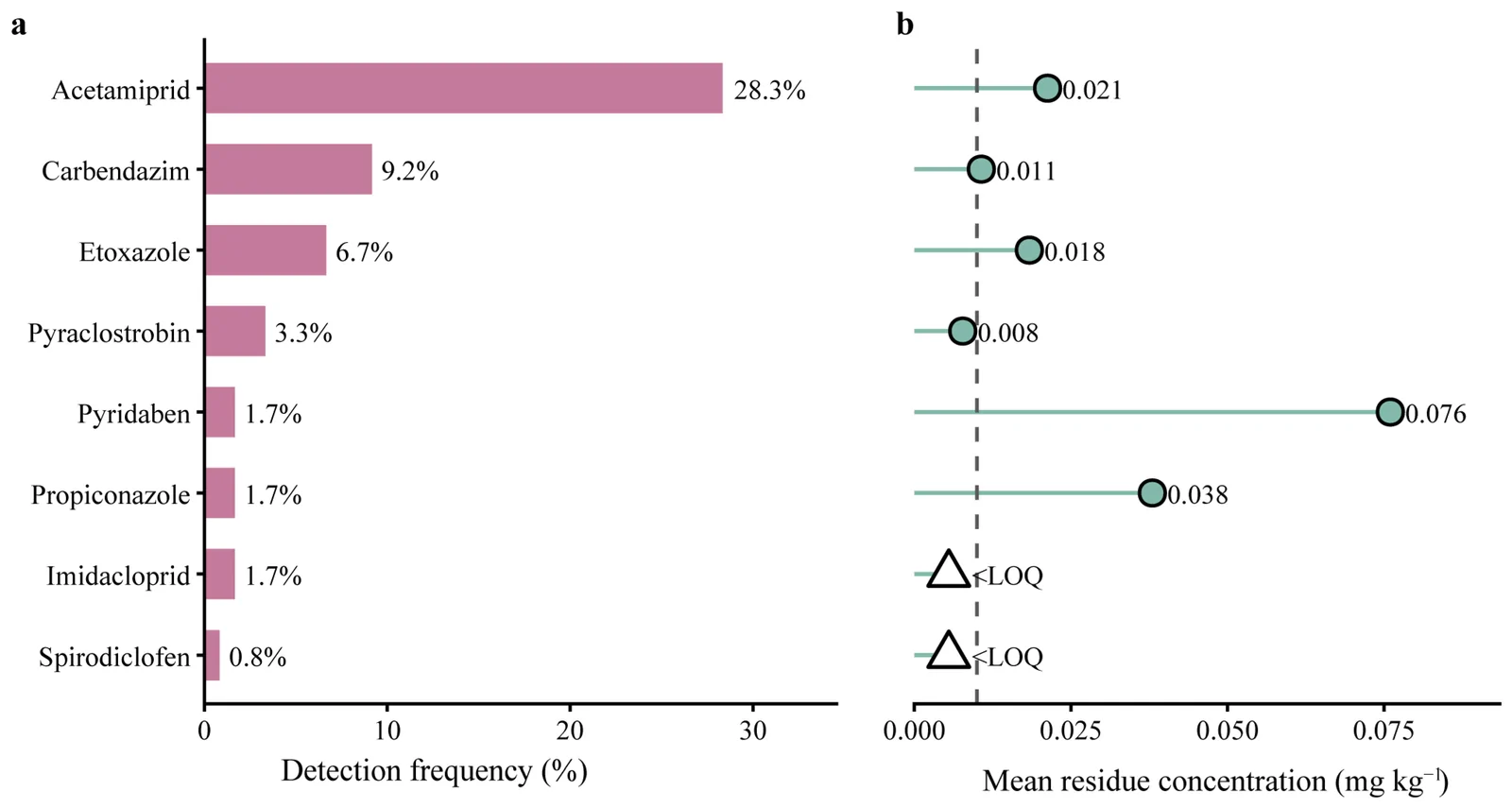
Detection rates and mean residue concentrations of the eight detected pesticides in positive apple samples from Xinjiang. (**a**) Detection frequency (%) of each pesticide. (**b**) Mean residue concentration (mg/kg) in positive samples; horizontal lines connect each mean value to the method limit of quantification (LOQ, dashed line, 0.01 mg/kg). Open triangles denote imidacloprid and spirodiclofen, whose instrument signals corresponded to estimated concentrations below the LOQ but within the calibration range.

In this study, 82 pesticides were screened in 120 apple samples collected from Xinjiang, and 8 pesticides were detected. Among the 120 samples, at least one pesticide was detected in 50 samples, yielding an overall detection rate of 41.67%. The detected pesticides included three fungicides (propiconazole, pyraclostrobin, and carbendazim), three acaricides (pyridaben, spirodiclofen, and etoxazole), and two insecticides (acetamiprid and imidacloprid). The detection rates and mean residue concentrations in positive samples for each pesticide are shown in Figure 2.

Acetamiprid had the highest detection rate, at 28.3%, while pyridaben showed the highest mean residue concentration in positive samples, at 0.076 mg/kg. The maximum residue concentration of acetamiprid was 0.170 mg/kg, recorded in a sample collected from Wensu County, Aksu Prefecture. According to GB 2763-2021, none of the quantified results exceeded the corresponding maximum residue limits (MRLs). Although imidacloprid and spirodiclofen produced identifiable instrument signals within the calibration range, the corresponding estimated concentrations were below the method limit of quantification (LOQ); therefore, they were not treated as confirmed quantitative results and were excluded from comparisons of mean residue concentrations. In the chronic exposure assessment, these data were treated under the LB and UB scenarios in accordance with the rules described in Section 2.4.

### 3.2. Multi-Pesticide Residue Occurrence in Apple Samples

Among the 120 apple samples, 70 (58.33%) contained no detectable pesticide residues, 39 (32.50%) contained a single pesticide, 9 (7.50%) contained two pesticides, and 1 sample each (0.83%) contained three and four pesticides, respectively. A total of 50 samples tested positive for at least one pesticide, giving a detection rate of 41.67%. Of these, 11 samples contained two or more pesticides, representing 9.17% of all samples and 22.00% of the positive samples. The distribution of pesticide residue counts per sample is shown in Table 3.

Regionally, multi-pesticide co-occurrence was concentrated in Aksu. Among the 65 samples from Aksu, 10 contained two or more pesticides (co-occurrence rate: 15.38%). In Kashgar, only 1 of 25 samples contained two pesticides (co-occurrence rate: 4.00%). All 30 samples from Ili Prefecture either had no detected pesticide residues or contained only a single pesticide, with no multi-pesticide co-occurrence observed. Compared with Ili and Kashgar, Aksu exhibited a higher co-occurrence rate and a broader range of pesticide combinations.

**Figure 3:**
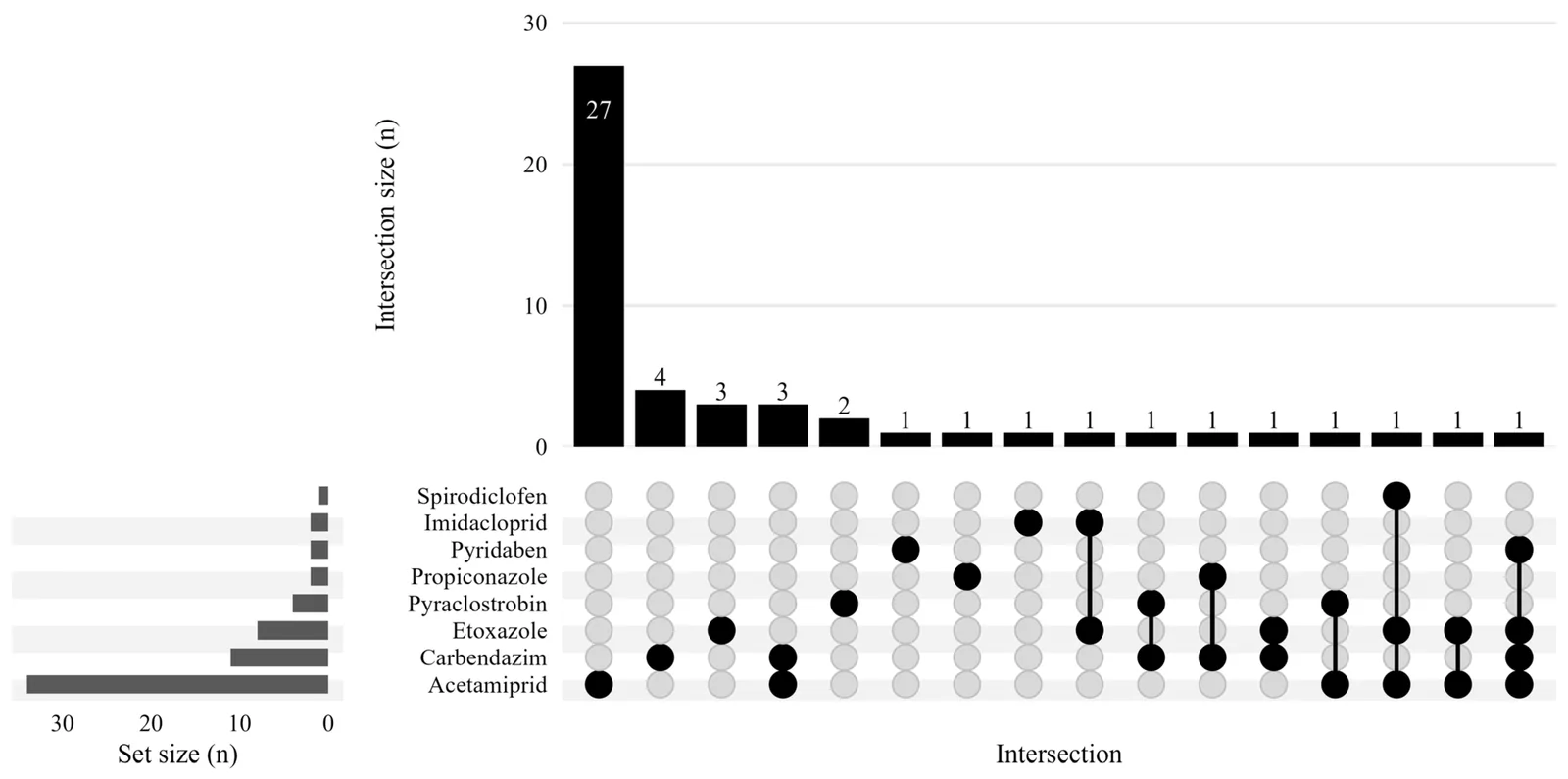
Distribution of pesticide occurrence and co-occurrence patterns in apple samples from Xinjiang. Black circles indicate the pesticides in each residue combination, and connecting lines indicate their co-occurrence.

In terms of specific combinations, “carbendazim + acetamiprid” was the most frequent binary combination, detected in three samples. Binary combinations of “acetamiprid + etoxazole,” “carbendazim + etoxazole,” “carbendazim + propiconazole,” “carbendazim + pyraclostrobin,” “acetamiprid + pyraclostrobin,” and “imidacloprid + etoxazole” each occurred in a single sample. One sample from Wensu County contained acetamiprid, spirodiclofen, and etoxazole simultaneously, forming a ternary combination. One sample from Hongqipo Farm contained carbendazim, acetamiprid, pyridaben, and etoxazole, forming a quaternary combination. The distribution of specific combinations is illustrated in Figure 3.

It should be noted that imidacloprid and spirodiclofen were included in the co-occurrence analysis based on their instrumental signals; however, their concentrations fell below the method quantification limits and were not reported as confirmatory quantitative results. The specific risks associated with multi-pesticide co-occurrence require further evaluation integrating residue concentrations, toxicological reference values, and dietary exposure parameters [27].

### 3.3. Spatial Variation in Pesticide Residues Among Production Regions

Pesticide occurrence varied among the three major apple-producing regions [28]. In Aksu, pesticide residues were detected in 34 of 65 samples, corresponding to a detection rate of 52.31%, compared with 26.67% (8/30) in Ili and 32.00% (8/25) in Kashgar. The overall detection rates differed significantly among the three regions (χ^2^ = 6.77, p = 0.034), with the highest rate observed in Aksu. Eight pesticides were detected in Aksu, representing all pesticide compounds identified in this study. By comparison, three pesticides—carbendazim, acetamiprid, and pyraclostrobin—were detected in Ili, whereas four—acetamiprid, imidacloprid, propiconazole, and etoxazole—were detected in Kashgar. Together with the co-occurrence results, these findings show that samples from Aksu had not only a higher overall detection rate but also a more diverse residue profile and a greater occurrence of multiple residues, indicating clear regional variation in pesticide residue patterns.

**Figure 4:**
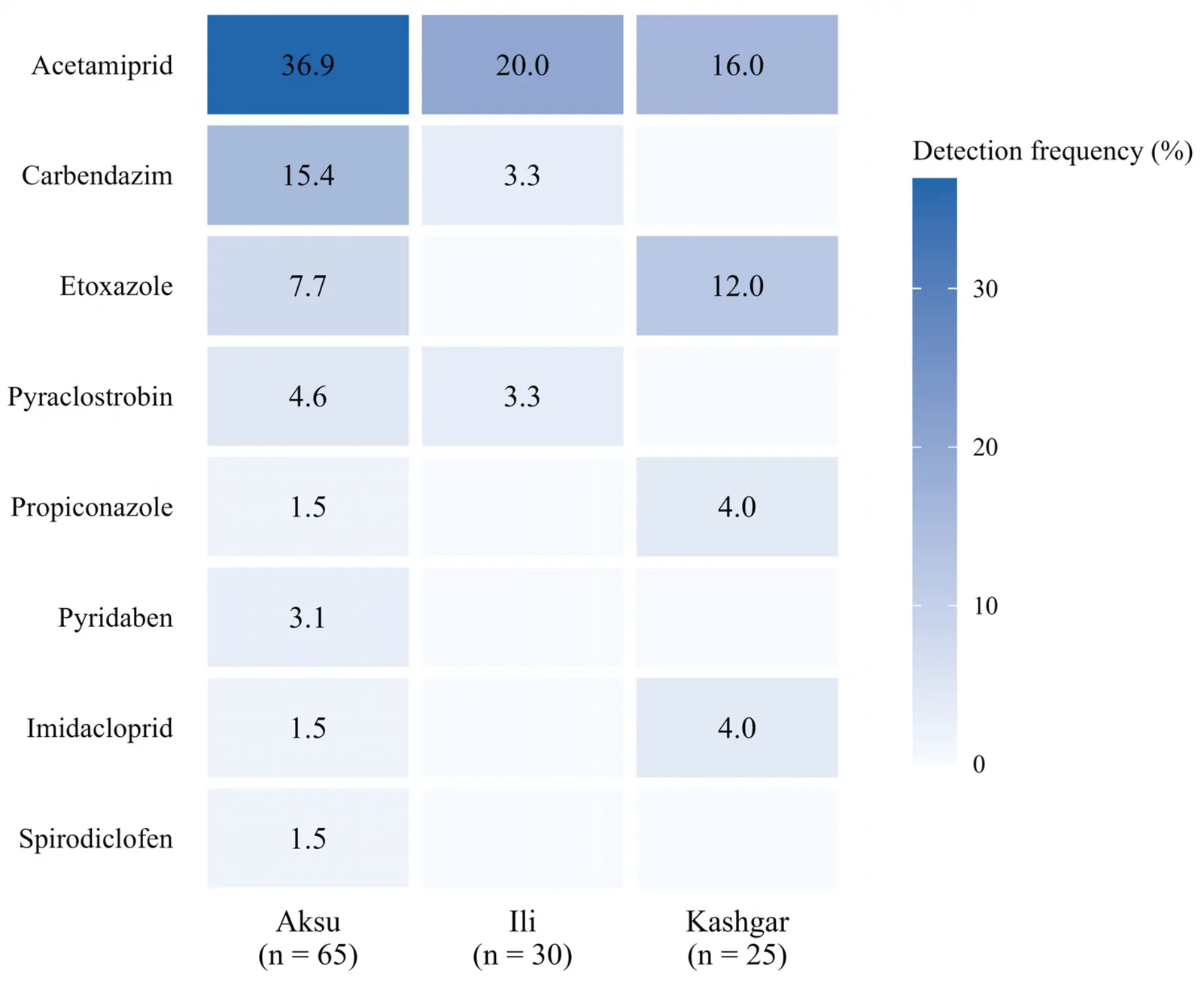
Detection frequencies of eight pesticides in apples from different production regions.

Detection frequencies by region are shown in Figure 4. Acetamiprid was the only pesticide detected in all three regions, although both its detection frequency and residue level differed among regions. Its detection rates were 36.92%, 20.00%, and 16.00% in Aksu, Ili, and Kashgar, respectively, while the corresponding maximum concentrations were 0.170, 0.054, and 0.012 mg/kg. Carbendazim was mainly detected in Aksu, with a detection rate of 15.38%; only one positive sample was found in Ili, and none was detected in Kashgar. Etoxazole occurred in both Aksu (7.69%) and Kashgar (12.00%) but was not detected in Ili. Pyridaben and spirodiclofen were detected only in Aksu, while pyraclostrobin was also mainly found in Aksu, with only one positive sample in Ili. Overall, Aksu showed a higher detection frequency and a more complex residue composition, whereas the residue profiles in Ili and Kashgar were relatively limited in diversity. However, because detailed records of pest occurrence and pesticide application were unavailable for the sampled orchards, the observed regional differences cannot be directly attributed to differences in pesticide use or orchard management. Further studies incorporating orchard-level pesticide application and pest management records are needed to clarify how regional differences in pest management and pesticide use contribute to the observed variation.

### 3.4. Chronic and Acute Dietary Exposure Risks in Different Age Groups

**Figure 5:**
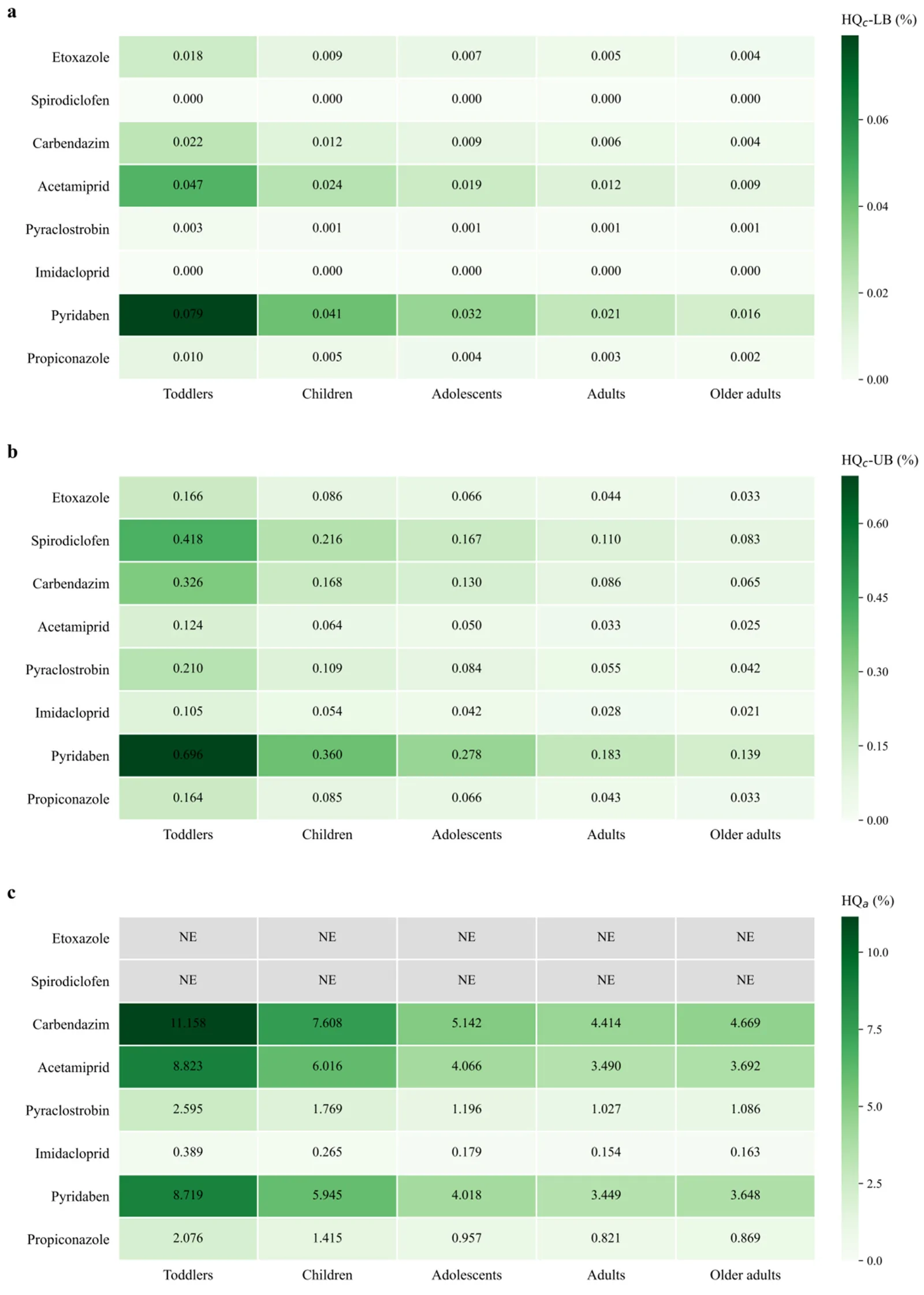
Age-specific dietary exposure risks of pesticide residues detected in apple samples under different exposure scenarios. (**a**) Chronic hazard quotient under the lower-bound scenario (HQ_c_-LB); (**b**) chronic hazard quotient under the upper-bound scenario (HQ_c_-UB); (**c**) acute hazard quotient (HQ_a_). NE indicates that HQ_a_ was not calculated for etoxazole or spirodiclofen because no numerical ARfD was available.

Chronic and acute hazard quotients across the five age groups are shown in Figure 5. Under both the lower-bound (LB) and upper-bound (UB) scenarios, chronic hazard quotients (HQ_c_) for the eight detected pesticides were below 100% across all five age groups. Toddlers had the highest chronic exposure levels. Within the toddler group, HQ_c_ values for the eight pesticides ranged from 0 to 0.079% under the LB scenario and from 0.105% to 0.696% under the UB scenario. Across the five age groups, HQ_c_ values generally decreased with increasing age. Pyridaben had the highest HQ_c_ under both scenarios, reaching 0.079% and 0.696% in toddlers under the LB and UB scenarios, respectively. The HQc values estimated under the UB scenario were consistently higher than those estimated under the LB scenario, reflecting the influence of left-censored data treatment on chronic exposure estimates. In no case did chronic exposure to any of the eight pesticides approach its acceptable daily intake (ADI) under either scenario.

In the acute exposure assessment, acute hazard quotients (HQ_a_) were not calculated for spirodiclofen or etoxazole because no numerical acute reference dose (ARfD) was available for either pesticide. The HQ_a_ values of the remaining six pesticides were below 100% in all age groups. Toddlers had the highest overall acute exposure levels. Within the toddler group, carbendazim had the highest HQ_a_, at 11.158%, followed by acetamiprid and pyridaben, at 8.823% and 8.719%, respectively. The HQ_a_ values of carbendazim in children, adolescents, adults, and older adults were 7.608%, 5.142%, 4.414%, and 4.669%, respectively. None of the six pesticides included in the acute assessment surpassed its ARfD in any age group.

Of the 80 pesticides analyzed in 2024, 33 were detected. Among these, 21 were also included in the 2025 analytical panel, including eight of the ten most frequently detected pesticides in 2024 (Table S5). The remaining 12 detected pesticides were assessed separately for dietary exposure in toddlers using the 2024 residue data (Table S4). Vamidothion had the highest chronic HQ under the upper-bound scenario (HQ_c_-UB, 0.786%), whereas fenpyroximate had the highest acute HQ (HQ_a_, 4.671%). All calculated HQs were below the 100% screening threshold.

### 3.5. Cumulative Dietary Exposure Risks and Risk Contributions

**Figure 6:**
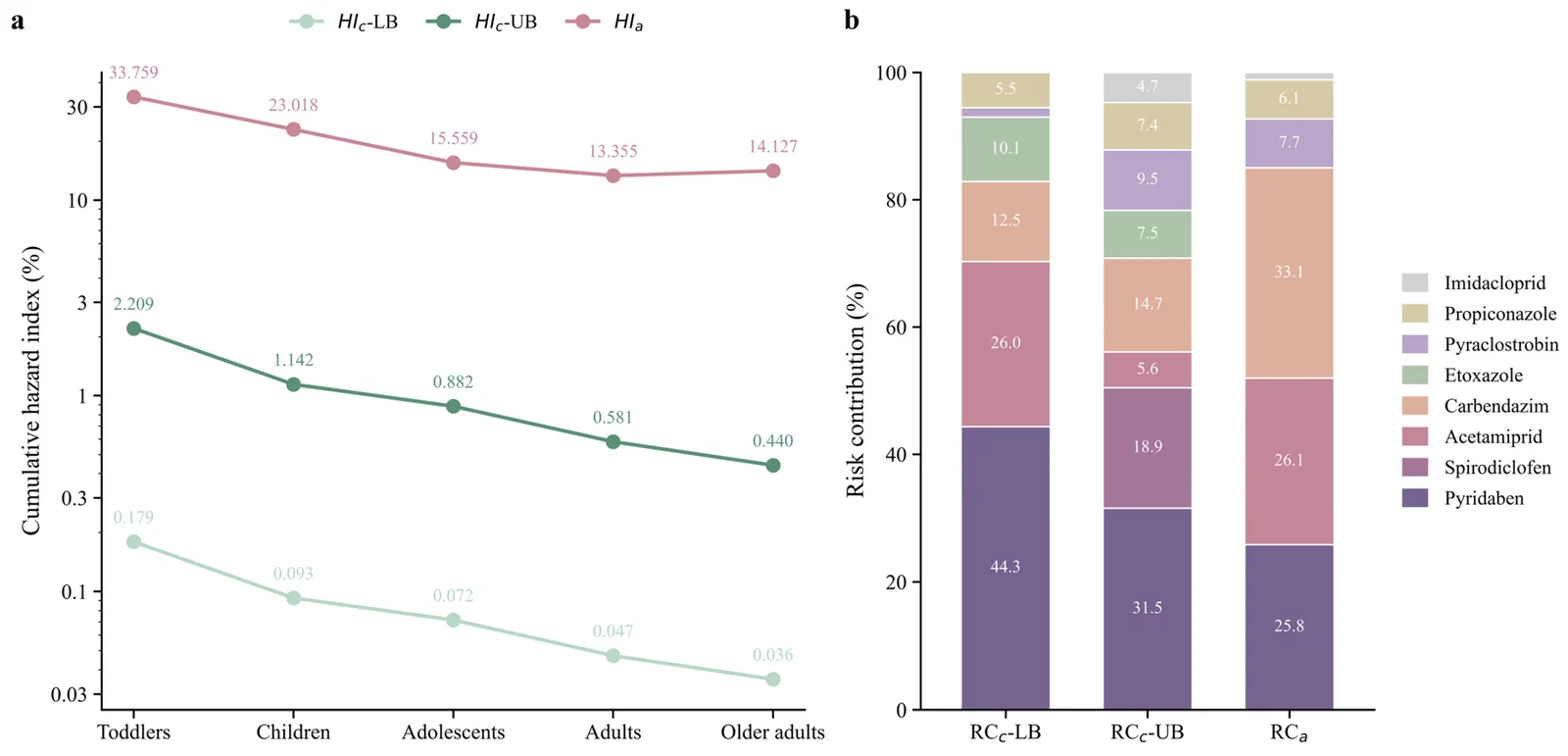
Age-specific cumulative dietary exposure risks and pesticide-specific risk contributions under different exposure scenarios. (**a**) Age-specific cumulative chronic hazard indices under the lower-bound (HI_c_-LB) and upper-bound (HI_c_-UB) scenarios, together with the cumulative acute hazard index (HI_a_). The y-axis in panel (**a**) is presented on a logarithmic scale. (**b**) Pesticide-specific contributions to cumulative chronic risk under the lower-bound (RCc-LB) and upper-bound (RCc-UB) scenarios and to cumulative acute risk (RCa) in toddlers. Etoxazole and spirodiclofen were excluded from the acute risk contribution analysis because no numerical acute reference doses were established. Contributions below 3% are not individually labeled.

The cumulative dietary exposure risks across age groups are presented in Figure 6. Under the LB scenario, the chronic cumulative hazard indices for toddlers, children, adolescents, adults, and older adults were 0.179%, 0.093%, 0.072%, 0.047%, and 0.036%, respectively; under the UB scenario, the corresponding values were 2.209%, 1.142%, 0.882%, 0.581%, and 0.440%. The acute cumulative hazard indices were 33.759%, 23.018%, 15.559%, 13.355%, and 14.127%, respectively. None of the cumulative hazard indices exceeded the 100% screening threshold in any age group. Toddlers had the highest cumulative exposure levels in both the chronic and acute assessments [29].

Taking toddlers as an example, pyridaben and acetamiprid accounted for 44.3% and 26.0% of the chronic cumulative risk under the LB scenario, respectively. Under the UB scenario, pyridaben, spirodiclofen, and carbendazim contributed 31.5%, 18.9%, and 14.7%, respectively. The main contributors to acute cumulative risk were carbendazim, acetamiprid, and pyridaben, with contribution rates of 33.05%, 26.13%, and 25.83%, respectively. Together, these three pesticides accounted for 85.0% of the acute cumulative risk.

A further comparison of the rankings based on detection frequency and chronic and acute risk contributions revealed clear discrepancies among the eight pesticides (Table 4). Acetamiprid ranked first in detection frequency but only seventh in chronic risk contribution under the UB scenario, although it ranked second in acute risk contribution. In contrast, pyridaben had a detection frequency of only 1.67% but ranked first in chronic risk contribution and third in acute risk contribution. Spirodiclofen had the lowest detection frequency, at 0.83%, yet ranked second in chronic risk contribution under the UB scenario. Carbendazim ranked second in detection frequency but was the leading contributor to acute cumulative risk. These shifts in rank indicate that detection frequency alone does not reliably identify the pesticides making the greatest contributions to dietary exposure risk. Risk contribution is also shaped by residue concentration, the corresponding ADI or ARfD, and age-specific food consumption and body weight.

### 3.6. Multi-Pesticide Co-Occurrence and Sample-Level Cumulative Risk Characteristics

Among the 120 apple samples, at least one pesticide was detected in 50 samples, of which 39 contained a single pesticide and 11 contained two or more. Based on the number of pesticides detected per sample, these samples were divided into a single-residue group (*n* = 39) and a multi-pesticide co-occurrence group (*n* = 11). After values below the LOQ and non-detects were assigned according to the LB and UB scenarios, the chronic hazard quotients of the eight evaluated pesticides were summed for each sample to obtain a sample-level screening chronic cumulative hazard index (HI_c, sample_). Because toddlers had the highest ratio of apple consumption to body weight and therefore the highest relative exposure, their consumption and mean body-weight parameters were used for all between-group comparisons and correlation analyses.

**Figure 7:**
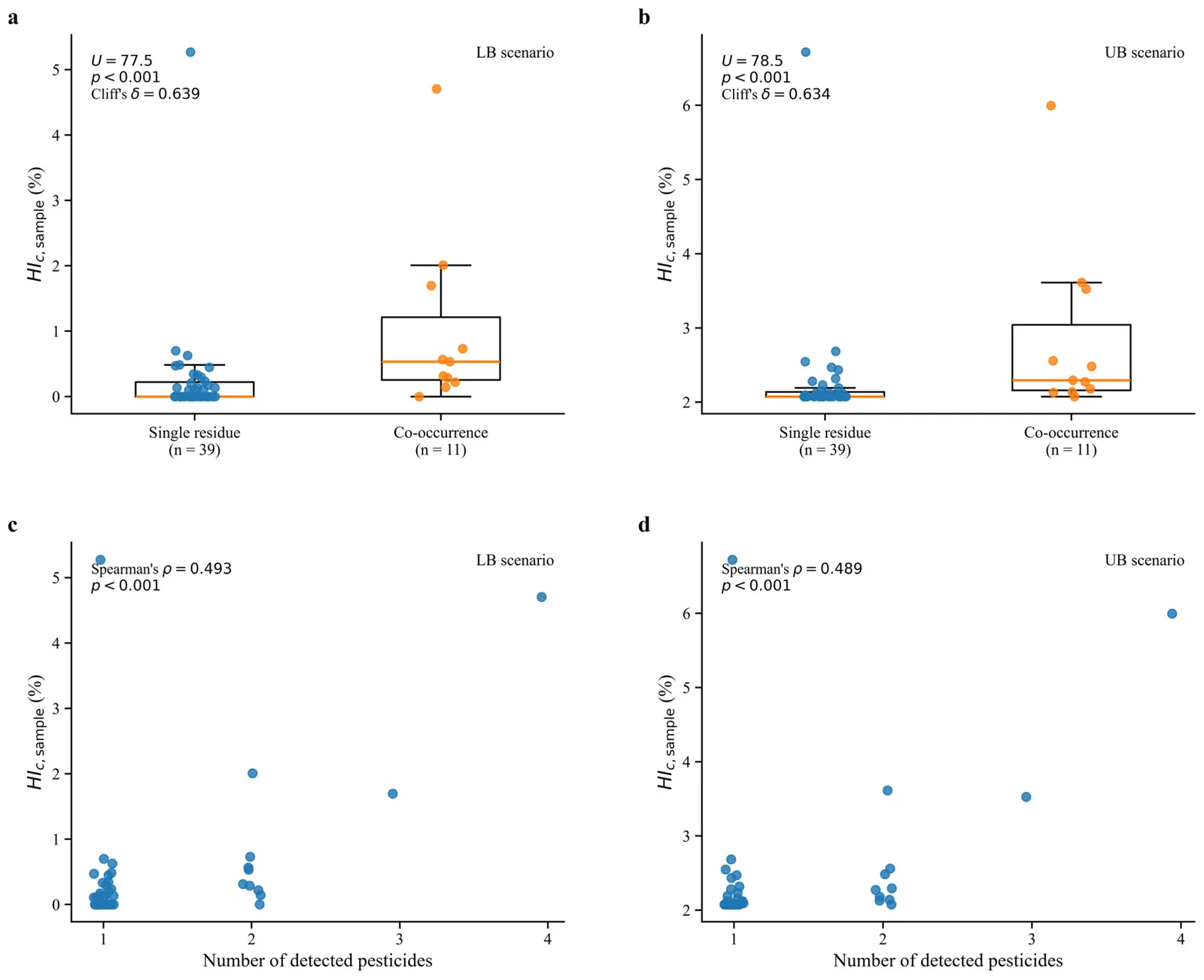
Pesticide co-occurrence and sample-level chronic cumulative risk. Panels (**a**,**b**) compare the sample-level chronic cumulative hazard index between the single-residue and co-occurrence groups under the LB and UB scenarios, respectively. Panels (**c**,**d**) show the relationships between the number of pesticide residues and the sample-level chronic cumulative hazard index under the LB and UB scenarios, respectively.

Under the LB scenario, the median HI_c, sample_ values were 0.000% in the single-residue group and 0.533% in the multi-pesticide co-occurrence group, with interquartile ranges of 0.000–0.221% and 0.253–1.213%, respectively. The Mann–Whitney U test showed a statistically significant difference between the two groups (U = 77.5, *p* = 0.0008, Cliff’s δ = 0.639). Under the UB scenario, the corresponding median values were 2.076% and 2.296%, with interquartile ranges of 2.076–2.138% and 2.160–3.043%, respectively; the difference between the two groups was likewise significant (U = 78.5, *p* = 0.0009, Cliff’s δ = 0.634). The large effect sizes observed under both scenarios indicated that the multi-pesticide co-occurrence group generally had higher sample-level chronic cumulative exposure than the single-residue group (Figure 7). None of the 50 samples exceeded the 100% screening threshold for HI_c, sample_.

Among the 50 samples with detected pesticides, the number of pesticides detected was significantly and positively correlated with HI_c,sample_ under both scenarios (LB: ρ = 0.493; UB: ρ = 0.489; both *p* < 0.001). However, the relationship was not strictly proportional. Some multi-pesticide co-occurrence samples had relatively low HI_c,sample_ values because the concentrations of the individual residues were low, whereas some single-residue samples showed relatively high risk indices owing to a high residue-to-ADI ratio. Thus, the number of co-occurring pesticides mainly reflects the complexity of the residue profile and cannot substitute for a quantitative risk assessment that integrates residue concentrations and toxicological reference values.

One illustrative sample was collected from the Yuanshengtai Linhai Base of Hongqipo Farm and contained carbendazim, acetamiprid, pyridaben, and etoxazole. The instrument signals for carbendazim and acetamiprid corresponded to concentrations of 0.004 mg/kg and 0.007 mg/kg, respectively, both of which were below the method LOQ. The residue concentrations of pyridaben and etoxazole were 0.068 mg/kg and 0.028 mg/kg, respectively. Under the LB scenario, the below-LOQ results for carbendazim and acetamiprid were assigned a value of zero. Under the UB scenario, all results below the LOQ and all non-detects were assigned the LOQ. The HI_c, sample_ values of this sample were 4.705% under the LB scenario and 5.996% under the UB scenario. It ranked second among the 50 samples with detected pesticides under both scenarios, surpassed only by a sample containing pyridaben at 0.084 mg/kg.

Under the LB scenario, pyridaben accounted for 90.7% of the HI_c, sample_ of this sample. Under the UB scenario, its contribution—calculated as the ratio of the pyridaben HQ_c_ to the sum of the HQ_c_ values for all eight evaluated pesticides—was 71.1%. Pyridaben therefore remained the predominant contributor to the chronic cumulative risk of this sample. Although none of the analytical results indicated an exceedance of the corresponding MRLs, the sample-level cumulative exposure was higher than that of most other samples, demonstrating that compliance with individual MRLs does not guarantee equivalent cumulative exposure across samples.

The sample-level HI_c_ calculated in this study was based on the dose-addition assumption and was intended primarily for comparing relative cumulative exposure among samples with different residue patterns. It cannot, by itself, support inferences of synergistic, antagonistic, or other confirmed combined toxic effects among pesticides [30].

### 3.7. Sensitivity Analysis of Toxicological Reference Values for Acetamiprid

The primary analysis used the toxicological reference values for acetamiprid established by JMPR, with an ADI of 0.07 mg/kg bw/day and an ARfD of 0.10 mg/kg bw [24]. Under these values, both the chronic and acute hazard quotients of acetamiprid remained below the 100% screening threshold in all age groups. A sensitivity analysis was subsequently conducted using the toxicological reference values updated by the EFSA in 2024 [25], with an ADI of 0.005 mg/kg bw/day and an ARfD of 0.005 mg/kg bw. Compared with the primary analysis, the chronic hazard quotient of acetamiprid increased 14-fold and the acute hazard quotient increased 20-fold. The acute hazard quotients for toddlers and children rose to 176.5% and 120.3%, respectively, both exceeding the 100% screening threshold.

With residue levels and dietary exposure parameters held constant, the selection and updating of toxicological reference values may substantially affect hazard quotients, risk-contribution rankings, and the determination of whether the screening threshold is exceeded. Therefore, the source and scope of applicability of the toxicological reference values used should be clearly stated when interpreting dietary pesticide exposure assessments.

## 4. Discussion

Eight of the 82 pesticides analyzed were detected in 120 apple samples collected from Aksu, Ili, and Kashgar, the three major apple-producing areas of Xinjiang. According to GB 2763-2021, all confirmed quantitative results fell below the corresponding maximum residue limits (MRLs). Acetamiprid had the highest detection frequency (28.3%), whereas pyridaben showed the highest mean concentration among positive samples (0.076 mg/kg), indicating that detection frequency does not necessarily correspond to residue level. Multi-pesticide co-occurrence was observed in 9.17% of the samples. Most co-occurrence cases were found in samples from Aksu. Compared with Ili and Kashgar, Aksu had a higher proportion of positive samples, a greater diversity of detected pesticides, and more co-occurrence patterns, suggesting regional heterogeneity in pesticide residue occurrence. However, because records of pesticide application, pest occurrence, and orchard management were unavailable, these differences cannot be directly attributed to pesticide-use intensity or specific management practices.

The overall detection rate of 41.67% was below the 58.6% reported for apples from other Chinese production regions [8] and the 91.7% recorded in market-sourced apples nationwide [31]. A detection rate of 64% was reported in Turkish apple samples [32], where acetamiprid was detected in 24% of samples—close to the 28.3% recorded in the present study—although multi-residue occurrence and MRL exceedances were more frequent there. A long-term Polish monitoring program reported a detection rate of 64.2%, with acetamiprid again among the frequently detected insecticides [33]. Pesticide co-occurrence has also been reported in apples from other countries and regions [32,34]. These findings place the present results within a broader monitoring context. However, differences in analytical scope, reporting limits, sampling design, and monitoring periods limit direct comparisons of detection frequencies across studies.

The dietary exposure assessment further showed that neither detection frequency nor residue concentrations in positive samples alone were sufficient for risk prioritization. Under both the lower-bound (LB) and upper-bound (UB) scenarios, the chronic hazard quotients (HQ_c_) of individual pesticides remained below 100% in all age groups. The chronic cumulative hazard indices (HI_c_) for toddlers were 0.179% and 2.209% under the LB and UB scenarios, respectively. Assigning different values to results below the limit of quantification (LOQ) affected the magnitude of the chronic exposure estimates without changing their overall screening interpretation. Pyridaben remained the leading contributor to HI_c_ under both scenarios, although its proportional contribution varied with the treatment of results below the LOQ. The consistently higher relative exposure in toddlers was associated with their higher ratio of apple consumption to body weight [35,36]. This age-related pattern is consistent with previous work on Polish apples, which estimated greater dietary risk in children than in adults [37]. Overall, dietary risk was influenced jointly by residue concentration, toxicological reference values, food consumption, and body weight [38].

For the acute assessment, summing the HQs derived from each pesticide’s highest observed residue concentration yielded an HI_a_ of 33.759% in toddlers. Because these maximum concentrations did not necessarily occur in the same sample, the summed value represents a conservative screening scenario rather than an observed co-exposure in a single sample. Carbendazim, acetamiprid, and pyridaben together accounted for 85.0% of the summed HI_a_. At the sample level, the chronic hazard index was significantly higher in the multi-pesticide co-occurrence group than in the single-residue group (*p* < 0.001), indicating greater screening-level cumulative exposure in samples containing multiple residues. The hazard indices were therefore interpreted only as screening-level indicators. Because the toxicological reference values for individual pesticides may be based on different critical effects, the summed indices should not be interpreted as direct measures of combined toxicity. The acute estimate for imidacloprid should also be interpreted with caution because its screening-level highest residue was derived from an instrument signal corresponding to a concentration below the method LOQ. Etoxazole and spirodiclofen were not included in the acute risk assessment because no numerical ARfD was available for either pesticide. Nevertheless, the observed pesticide co-occurrence supports consideration of combined exposure alongside the assessment of individual compounds, an approach that has also been applied in risk assessments of apples from the Greek market [34].

MRL compliance and dietary risk assessment serve different purposes [32], as illustrated by the sensitivity analysis of acetamiprid. Under the JMPR reference values adopted in the primary analysis, the chronic and acute hazard quotients of acetamiprid remained below 100% in all age groups. When the updated EFSA 2024 reference values were applied, its chronic HQ increased markedly, while the acute HQs in toddlers and children reached 176.5% and 120.3%, respectively, exceeding the 100% screening threshold. The residue and consumption inputs were unchanged; the differences in the resulting HQs arose from the toxicological reference values applied. These findings demonstrate that dietary risk interpretation depends not only on measured residues and exposure parameters but also on the toxicological benchmarks selected for assessment. The source, version, and scope of applicability of toxicological reference values should therefore be clearly stated when interpreting dietary exposure assessments.

Pesticide selection for the 2025 monitoring program was informed by previous regional monitoring results, the applicability of multiresidue analytical methods, and annual surveillance priorities. Of the 33 pesticides detected in the 2024 monitoring dataset, 21 were also included in the 2025 analytical panel, including eight of the ten most frequently detected compounds (Table S5). For the remaining 12 pesticides, a supplementary dietary exposure assessment using the 2024 residue data showed that all calculated individual HQs were below the 100% screening threshold (Table S4). The highest HQ_c_-UB was 0.786% for vamidothion, whereas the highest HQ_a_ was 4.671% for fenpyroximate. These findings were consistent with the generally low dietary risk estimated in the primary 2025 assessment and provided complementary evidence from the preceding monitoring year.

Several limitations should be considered. The primary 2025 dataset was derived from a single sampling campaign in Aksu, Ili, and Kashgar and therefore cannot fully capture seasonal variation or broader spatial heterogeneity in pesticide residues across Xinjiang. The separately assessed 2024 data provide useful historical context but are not sufficient to establish temporal trends.

Under the primary assessment framework, the dietary risks associated with the evaluated pesticide residues in apples were generally low. However, applying the updated EFSA reference values for acetamiprid resulted in acute HQs above the screening threshold in toddlers and children. Taken together, these findings indicate that monitoring priorities should not be based solely on detection frequency or MRL compliance. Residue concentrations, age-specific exposure, pesticide co-occurrence, and updated toxicological reference values should also be considered in risk-based monitoring of pesticide residues in apples.

## Figures and Tables

**Table 1:** Age-specific body weight and apple consumption parameters used in the dietary exposure assessment [17].

Population Group	Age (Years)	Mean Body Weight (kg)	Mean Consumption (kg/Day)	Large-Portion Consumption (kg/Day)	Mean Unit Weight of an Apple (kg)
Toddlers	2–6	17.6	0.1104	0.4034	0.2550
Children	7–13	34.0	0.1103	0.6931	0.2550
Adolescents	14–17	50.3	0.1260	0.6931	0.2550
Adults	18–59	58.6	0.0967	0.6931	0.2550
Older adults	≥60	55.4	0.0693	0.6931	0.2550

**Table 2:** Acceptable daily intakes, acute reference doses, and sources for the eight detected pesticides.

Pesticide	ADI (mg/kg bw/Day)	ARfD (mg/kg bw)	Source
Acetamiprid	0.07	0.10	JMPR
Carbendazim	0.02	0.02	EFSA
Pyridaben	0.01	0.05	EFSA
Etoxazole	0.04	NE	EFSA
Spirodiclofen	0.015	NE	EFSA
Pyraclostrobin	0.03	0.03	EFSA
Propiconazole	0.04	0.10	EFSA
Imidacloprid	0.06	0.08	EFSA

**Table 3:** Distribution of the number of pesticide residues detected in apple samples.

Number of Pesticides Detected per Sample	Number of Samples	Proportion of Total Samples (%)
0	70	58.33
1	39	32.50
2	9	7.50
3	1	0.83
4	1	0.83
≥2	11	9.17

**Table 4:** Rankings of detection frequencies and contributions to chronic and acute dietary risks for the eight pesticides.

Pesticide	Detection Frequency Rank	Detection Frequency (%)	Chronic Risk Contribution Rank (UB)	Chronic Risk Contribution (%)	Acute Risk Contribution Rank	Acute Risk Contribution (%)
Acetamiprid	1	28.33%	7	5.6%	2	26.13%
Carbendazim	2	9.17%	3	14.7%	1	33.05%
Etoxazole	3	6.67%	5	7.5%	NE	/
Pyraclostrobin	4	3.33%	4	9.5%	4	7.69%
Propiconazole	5 *	1.67%	6	7.4%	5	6.15%
Pyridaben	6 *	1.67%	1	31.5%	3	25.83%
Imidacloprid	7 *	1.67%	8	4.7%	6	1.15%
Spirodiclofen	8	0.83%	2	18.9%	NE	/

Note: * Propiconazole, pyridaben, and imidacloprid were each detected in 2 of 120 samples (1.67%). Their detection-frequency ranks are shown sequentially for presentation only and do not indicate a strict priority. NE, not evaluated because no numerical ARfD was available.

## Data Availability

The data presented in this study are available in the article and its Supplementary Materials.

## Supplementary Materials

The following supporting information can be downloaded at https://www.mdpi.com/article/10.3390/foods15193491/s1, Table S1: Audit of JMPR availability, JMPR–EFSA consistency, and toxicological reference values used for the eight detected pesticides; Table S2: Comparison of maximum residue limits (MRLs) for the eight detected pesticides between GB 2763-2021 [15] and GB 2763-2026 [39]; Table S3: Instrumental parameters; Table S4: Supplementary dietary exposure assessment based on 2024 monitoring data. Table S5: Summary of the 2024 pesticide monitoring results for Xinjiang apples and coverage of these pesticides in the 2025 monitoring panel.

## Abbreviations

Acronym	Definition
ADI	Acceptable Daily Intake
ARfD	Acute Reference Dose
EDI	Estimated Daily Intake
EFSA	European Food Safety Authority
GC–MS/MS	Gas Chromatography–Tandem Mass Spectrometry
GC–ECD	Gas Chromatography with Electron Capture Detection
HI_c_	Chronic Cumulative Hazard Index
HI_a_	Acute Cumulative Hazard Index
HQ_c_	Chronic Hazard Quotient
HQ_a_	Acute Hazard Quotient
HR	Highest Residue
IQR	Interquartile Range
JMPR	Joint FAO/WHO Meeting on Pesticide Residues
LB	Lower Bound
LC–MS/MS	Liquid Chromatography–Tandem Mass Spectrometry
LOQ	Limit of Quantification
LP	Large Portion
MRL	Maximum Residue Limit
MRM	Multiple Reaction Monitoring
NE	Not Evaluated
PSA	Primary Secondary Amine
QuEChERS	Quick, Easy, Cheap, Effective, Rugged, and Safe
RCc	Chronic Risk Contribution
RCa	Acute Risk Contribution
RSD	Relative Standard Deviation
UB	Upper Bound
