# Supplementary material for: Multi-Pesticide Residue Occurrence and Age-Specific Dietary Exposure Risk Assessment of Apples from Major Production Areas in Xinjiang, China
Source: Foods. 2026 Sep 29;15(19):3491. doi: 10.3390/foods15193491 (PMC13665483; doi:10.3390/foods15193491)
Supplement: Supplementary file 1 [file foods-15-03491-s001.zip › Table S1 and S2.pdf]

Supplementary Materials

Pesticide Residues and Age-Specific Dietary Exposure Risk of Apples from Xinjiang's Main Production Areas

Rationale for the Selection of Toxicological Reference Values

For each of the eight detected pesticides, the toxicological reference values (ADI and ARfD) used in this study were selected according to the current availability and consistency of JMPR and EFSA evaluations. For carbendazim, pyridaben, etoxazole, and spirodiclofen, no current JMPR value was available (carbendazim's JMPR evaluation having been withdrawn in 2023, and the other three never having been evaluated by JMPR), so EFSA values were adopted by default. For pyraclostrobin, propiconazole, and imidacloprid, JMPR and EFSA values were cross-checked against the Pesticide Properties DataBase (PPDB) and found to show no material divergence; EFSA values were used for consistency with the majority of the dataset. Acetamiprid was the only pesticide for which JMPR and EFSA values diverged substantially (JMPR: ADI = 0.07 mg/kg bw/day, ARfD = 0.10 mg/kg bw; EFSA, following its 2024 statement: ADI = ARfD = 0.005 mg/kg bw). The JMPR value was adopted as the primary reference for acetamiprid because it remained the current international benchmark at the time of writing and is consistent with the ADI specified in China's national standard GB 2763—2021. Given the magnitude of the divergence between the two sources, the potential impact of adopting the updated EFSA value was explored separately through the sensitivity analysis reported in Section 3.7 of the main text. A full audit of JMPR availability and JMPR–EFSA consistency for all eight pesticides is provided in Table S1.

Table S1: Audit of JMPR availability, JMPR–EFSA consistency, and toxicological reference values used for the eight detected pesticides.

**Pesticide**	**JMPR status**	**JMPR–EFSA consistency**	**Reference value used in this study**
Acetamiprid	Current	Substantial divergence from EFSA (ADI 0.07 vs 0.005 mg/kg bw/day); addressed via sensitivity analysis	JMPR
Carbendazim	Withdrawn (2023)	Not applicable — no current JMPR value	EFSA
Pyridaben	Never evaluated	Not applicable — no JMPR value exists	EFSA
Etoxazole	Never evaluated	Not applicable — no JMPR value exists	EFSA
Spirodiclofen	Never evaluated	Not applicable — no JMPR value exists	EFSA
Pyraclostrobin	Current	No material divergence from EFSA (cross-checked via PPDB)	EFSA
Propiconazole	Current	No material divergence from EFSA	EFSA

		(cross-checked via PPDB)	
Imidacloprid	Current	No material divergence from EFSA (cross-checked via PPDB)	EFSA

Comparison of MRLs Between GB 2763-2021 and GB 2763-2026

Apple samples analyzed in this study were collected in October 2025, while GB 2763-2021 was the current national standard for maximum residue limits (MRLs) in China. GB 2763-2026 subsequently took effect on 1 March 2026, after sample collection had concluded. Because the revised standard could, in principle, alter the MRL-compliance conclusions reported in the main text, the MRLs specified for all eight detected pesticides were individually checked against both the 2021 and 2026 versions of the standard. As shown in Table S2, the MRL for each of the eight pesticides is identical between the two versions; no revisions relevant to this study were introduced by the 2026 update. The MRL-compliance conclusions presented in the main text are therefore unaffected by the timing of the standard's revision.

Table S2: Comparison of maximum residue limits (MRLs) for the eight detected pesticides between GB 2763-2021 [15] and GB 2763-2026 [39].

**Pesticide**	**MRL under GB 2763-2021 [15] (mg/kg)**	**MRL under GB 2763-2026 [39] (mg/kg)**
Acetamiprid	0.8	0.8
Carbendazim	5	5
Etoxazole	0.1	0.1
Pyraclostrobin	0.5	0.5
Propiconazole	0.1	0.1
Pyridaben	2	2
Imidacloprid	0.5	0.5
Spirodiclofen	0.5	0.5

References:

15. *GB 2763-2021*; National Food Safety Standard—Maximum Residue Limits for Pesticides in Food. Standards Press of China: Beijing, China, 2021.

39. *GB 2763-2026*; National Food Safety Standard—Maximum Residue Limits for Pesticides in Food. Standards Press of China: Beijing, China, 2026,
