# Supplementary material for: Multi-Pesticide Residue Occurrence and Age-Specific Dietary Exposure Risk Assessment of Apples from Major Production Areas in Xinjiang, China
Source: Foods. 2026 Sep 29;15(19):3491. doi: 10.3390/foods15193491 (PMC13665483; doi:10.3390/foods15193491)
Supplement: Supplementary file 1 [file foods-15-03491-s001.zip › 2025Xinjiang Monitoring Notice-English.pdf]

English translation provided for reference. The Chinese original is the source document.

Forestry and Grassland Administration of the Xinjiang Uygur Autonomous Region

Xin Lin Ban Han [2025] No. 198

Notice on Conducting the 2025 Quality and Safety Monitoring of Edible Forest Products and Soils in Their Production Areas in the Autonomous Region

To the forestry and grassland administrations of all prefectures, autonomous prefectures and cities:

In accordance with the Notice of the General Office of the National Forestry and Grassland Administration on Issuing the 2023-2025 Provincial Work Plan for Monitoring the Quality and Safety of Edible Forest Products (Ban Kexue [2023] No. 68), and in light of the actual circumstances of such monitoring in Xinjiang, we have prepared the 2025 Plan for Monitoring the Quality and Safety of Edible Forest Products and Soils in Their Production Areas in the Autonomous Region (see the attachment; hereinafter the Plan). The Plan is hereby issued to you, together with the following requirements.

I. Cooperate in implementing the monitoring work

Local forestry and grassland authorities shall strictly follow the Plan, communicate proactively with the technical support organization, and ensure that monitoring proceeds in an orderly manner and is completed on time and to the required quality standards. They shall urge all inspected entities to fulfill their primary responsibility as producers and business operators for product quality and safety, and to cooperate actively with sampling and monitoring.

II. Standardize monitoring procedures

Local forestry and grassland authorities shall strengthen the coordination of monitoring, regulate the conduct of personnel, and standardize the selection of sampling areas, sample collection and sample transport to ensure scientific rigor, impartiality, authenticity and accuracy. If the technical support organization identifies problems, it shall promptly inform the relevant local forestry and grassland authorities and report the circumstances to the Forestry and Grassland Administration of the Autonomous Region.

III. Improve local monitoring capacity

In addition to the forest-fruit quality and safety monitoring organized by the Forestry and Grassland Administration of the Autonomous Region, authorities responsible for the forest-fruit sector at all levels are encouraged, taking account of local sectoral development,

to organize their own forest-fruit product quality and safety monitoring through qualified scientific research and testing organizations, thereby expanding local coverage in terms of monitored indicators, tree species and geographical areas.

Each organization shall implement these requirements in light of its own circumstances and, by 25 April, provide the details of its designated personnel (organization, name, position and professional title, telephone number and email address) to the technical support organization, the Forest and Grassland Resources Research Center of the Xinjiang Academy of Forestry Sciences.

Contact: Yang Lu
Telephone: 0991-4649261; 18899161201
Email: lycszx_office@163.com
Postal address: Xinjiang Academy of Forestry Sciences, No. 191 Anju South Road, Shuimogou District, Urumqi
Postal code: 830000

Attachment: 2025 Plan for Monitoring the Quality and Safety of Edible Forest Products and Soils in Their Production Areas in the Autonomous Region

Forestry and Grassland Administration of the Autonomous Region
22 April 2025

Copy to: Xinjiang Academy of Forestry Sciences.
Internal distribution: Deputy Director Yu; General Office; Science and Technology Division.
Issued by the Office of the Forestry and Grassland Administration of the Xinjiang Uygur Autonomous Region on 22 April 2025.

Attachment

2025 Plan for Monitoring the Quality and Safety of Edible Forest Products and Soils in Their Production Areas in the Autonomous Region

I. Products to be monitored

The edible forest products to be monitored comprise fresh produce from 12 categories of forest-fruit products: walnuts, red jujubes, apricots, apples, fragrant pears, grapes, almonds, goji berries, peaches, pomegranates, Xinmei plums and sea buckthorn; and one forest-derived medicinal material, Cistanche (roucongrong).

Production-area soils to be monitored are those from the production areas of walnuts, red jujubes, apricots, apples, fragrant pears, grapes, almonds, goji berries, peaches, pomegranates, Xinmei plums, sea buckthorn and Cistanche.

II. Monitoring areas and quantities

Monitoring shall cover large-scale plantations and individually operated orchards within key counties, county-level cities and districts producing edible forest products. Sampling locations shall cover at least three villages in each county, city or district. The specific monitoring areas, quantities and testing organizations are indicated in Appendix Table 1.

A total of 2,200 batches are to be monitored in 2025. (1) Edible forest products: 1,100 batches, comprising walnuts (185), red jujubes (180), apricots (205), apples (120), fragrant pears (90), grapes (90), almonds (30), goji berries (20), peaches (25), pomegranates (20), Xinmei plums (70), sea buckthorn (35) and Cistanche (30). (2) Soils from edible forest-product production areas: 1,100 batches,

comprising soils from walnut (185), red jujube (180), apricot (205), apple (120), fragrant pear (90), grape (90), almond (30), goji berry (20), peach (25), pomegranate (20), Xinmei plum (70), sea buckthorn (35) and Cistanche (30) production areas. Specific monitoring areas and quantities are provided in Appendix Table 1.

III. Sampling

(1) Sampling areas

Samples shall be collected from large-scale plantations, cooperatives, individually operated orchards and organic plantations. Monitoring sites shall be representative and located in the main production areas. In principle, no more than five samples shall be collected from each sampling site or area, which shall have a cultivated area of at least 50 mu. Edible forest-product samples and production-area soil samples shall correspond to one another. The samples shall reflect the local quality and safety of the monitored products and their production-area soils.

(2) Sampling requirements

The forestry and grassland authorities of the monitored cities, counties and districts shall provide a list of representative production bases according to local forest-fruit production. The testing organization shall select bases randomly from this list, and sampling personnel from the local forestry and grassland authorities shall independently collect samples and send them to the testing organization. The testing organization shall also randomly select monitored cities, counties or districts and collect samples jointly with local sampling personnel. Sampling sites shall be representative of local forest-fruit product quality and safety. Designated sampling bases shall not refuse inspection; otherwise, their products shall be treated as non-compliant.

To facilitate subsequent tracing of the source of problematic products, sampling personnel must complete the Sampling Form for Quality and Safety Monitoring of Edible Forest Products strictly in accordance with its requirements and obtain the seal and signature of the inspected organization or individual.

(3) Stage of sampling

Production-base stage: the principal production bases for the products selected for inspection in each monitored county or city.

(4) Sampling methods

Edible forest products shall be sampled with reference to the Technical Regulations for Quality and Safety Monitoring of Economic Forest Products (LY/T 2800-2017). Fresh-product sampling: walnuts, red jujubes, apricots, apples, fragrant pears, grapes, almonds, goji berries, peaches, pomegranates, Xinmei plums, sea buckthorn and Cistanche shall be sampled at maturity from production bases. Each sampled product shall be divided into two portions, one for initial testing and one for retesting; the specific quantities are given in Appendix Table 2. Samples shall be placed in clean, uncontaminated containers or plastic bags. Packaged samples shall be sealed, and both the sampling personnel and a representative of the inspected entity shall sign the seal.

Soil sampling shall follow the Technical Specifications for Soil Environmental Monitoring (HJ/T 166-2004). Soil shall be collected from a depth of 0-40 cm. The soil sample from each sampling area shall be a composite of material collected at no fewer than five points. The composite sample shall weigh at least 1 kg and shall be placed in a clean, uncontaminated container or plastic bag. Soil from the sampling points shall be thoroughly mixed before packaging. The package shall be sealed, and the sampling personnel and a representative of the inspected entity shall sign the seal.

(5) Size of the lot sampled

The sampling lot shall consist of products harvested in the same batch and produced in accordance with the production-management requirements of the base. In principle, the lot shall weigh at least 200 kg.

(6) Sample quantities

The specific sampling quantities for each product category are given in Appendix Table 2.

(7) Sample transport and storage

During transport and storage, both initial-test and retest samples of edible forest products shall be protected against contamination and deterioration and shall not be exposed to moisture, rain or direct sunlight. On receipt, the testing organization shall inspect the condition of the samples, including the integrity of seals and packaging. Damaged or deteriorated samples shall be collected again. Accepted samples shall be processed promptly. Initial-test samples shall be prepared and analyzed in accordance with the applicable analytical method standards; retest samples shall be stored frozen in their original condition. Production-area soil samples shall be securely packaged to prevent sample loss during transport. On receipt, the testing organization shall inspect the soil samples, including the integrity of seals and packaging. Where packaging has been damaged, sampling shall be repeated. Soil samples shall be prepared and analyzed in accordance with the applicable analytical method standards.

(8) Sampling personnel

Forestry and grassland authorities at all levels shall assign sampling personnel. Sampling personnel must carry valid identification, inspection authorization documents, notebooks, sampling forms and survey forms.

IV. Monitoring items and analytical standards

The monitoring items and analytical standards are given in Appendix Tables 3 and 4.

V. Assessment criteria and principles

Results shall be assessed against the limits in the National Food Safety Standard - Maximum Levels of Contaminants in Foods (GB 2762-2022) and the National Food Safety Standard - Maximum Residue Limits for Pesticides in Food (GB 2763-2021). If all monitored items comply, the conclusion shall be:

"The tested items for product XX sampled in this monitoring programme meet the limit requirements of standard XX." If one or more items fail to comply and remain non-compliant after retesting in accordance with the relevant standard, the conclusion shall be: "Test item XX for product XX sampled in this monitoring programme does not meet the limit requirements of standard XX; the other tested items meet the limit requirements of standard XX."

Production-area soil monitoring results shall be assessed against the risk screening values and risk intervention values in Soil Environmental Quality - Risk Control Standard for Soil Contamination of Agricultural Land (Trial) (GB 15618-2018). Specifically, if all tested items comply, the conclusion shall be: "The tested items for this production-area soil meet the risk screening value requirements of GB 15618-2018." If one or more items fail to comply and remain non-compliant after retesting as specified in the standard, the conclusion shall be: "The tested item(s) for this production-area soil do not meet the risk screening value requirements of GB 15618-2018; the other tested items meet the risk screening value requirements of GB 15618-2018."

VI. Data submission

After testing has been completed, the testing organization shall promptly compile the data and complete the data summary forms. Numerical results shall be rounded to the number of digits specified in the applicable analytical standard. Where the standard does not specify the number of digits, results shall be reported to three significant figures. For items not detected, the testing organization shall provide the detection limit of the corresponding analytical method. The testing organization shall also conduct a comprehensive assessment of the monitoring results and analyze their causes, and submit the findings to the higher-level authority as an annual report on the quality of edible forest products in the Autonomous Region.

VII. Schedule

Monitoring, sampling and testing shall be scheduled according to the maturity of the edible forest products being monitored.

In general, sampling and testing shall be completed in the month in which the edible forest product reaches maturity. The specific schedule is as follows.

By 30 June 2025: complete sampling and testing of apricots and soils from their production areas.

By 30 July 2025: complete sampling and testing of peaches, goji berries, almonds and soils from their production areas.

By 30 August 2025: complete sampling and testing of early- and mid-season grapes, Xinmei plums and soils from their production areas.

By 30 September 2025: complete sampling and testing of late-season grapes, fragrant pears, walnuts, sea buckthorn and soils from their production areas.

By 30 October 2025: complete sampling and testing of apples, pomegranates, red jujubes, Cistanche and soils from their production areas.

On 30 November 2025: testing organizations shall complete the collection and compilation of all documentation and submit the materials relating to the monitoring work to the Forestry and Grassland Administration of the Autonomous Region.

VIII. Work requirements

(1) Monitoring shall be conducted in accordance with the Work Specifications for Quality and Safety Monitoring of Edible Forest Products (2020 edition).

(2) Monitoring shall maintain scientific rigor, representativeness and authenticity.

(3) Forestry and grassland authorities, sampling organizations, testing organizations and their personnel involved in sampling shall collect samples, conduct tests and report results in accordance with the law. Sampling locations and sample information shall not be changed arbitrarily. Test data shall not be concealed, falsified or omitted. Results shall be authentic, objective and accurate.

(4) No organization or individual may publish the monitoring results without permission from the Forestry and Grassland Administration of the Autonomous Region.

(5) Following collection, samples shall be sent promptly to the laboratory. Contamination and deterioration shall be prevented during transport.

Appendices

1. 2025 Quality and Safety Monitoring Schedule for Edible Forest Products and Soils in Their Production Areas
2. Sample Quantities
3. 2025 Quality and Safety Monitoring Items and Applicable Standards for Edible Forest Products
4. 2025 Quality and Safety Monitoring Items and Applicable Standards for Production-Area Soils

Appendix Table 1

2025 Quality and Safety Monitoring Schedule for Edible Forest Products and Soils in Their Production Areas

Walnuts | Monitoring time: September

Prefecture / city	Product batches	Soil batches	County/site allocation for each sample type
Aksu	75	75	Aksu City 15; Wensu County 30; Kuqa City 10; Xinhe County 10; Wushi County 10
Kashgar	60	60	Zepu County 15; Shache County 15; Yecheng County 20; Maigaiti County 5; Bachu County 5
Hotan	50	50	Hotan County 10; Moyu County 15; Pishan County 5; Luopu County 5; Yutian County 5; Minfeng County 10
Total	185	185	

Red jujubes | Monitoring time: October

Prefecture / city	Product batches	Soil batches	County/site allocation for each sample type
Hami	5	5	Yizhou District 5
Bayingolin	30	30	Ruoqiang County 20; Qiemo County 10
Aksu	30	30	Aksu City 15; Wensu County 15
Kashgar	60	60	Zepu County 10; Maigaiti County 30; Yuepuhu County 10; Bachu County 10
Hotan	55	55	Hotan County 15; Luopu County 15; Cele County 15; Moyu County 10
Total	180	180	

Appendix Table 1

2025 Quality and Safety Monitoring Schedule for Edible Forest Products and Soils in Their Production Areas

Apricots | Monitoring time: May in Turpan; June and July elsewhere

Prefecture / city	Product batches	Soil batches	County/site allocation for each sample type
Turpan	30	30	Gaochang District 10; Tuokexun County 20
Bayingolin	25	25	Luntai County 20; Heshuo County 5
Aksu	20	20	Kuqa City 15; Wensu County 5
Kashgar	60	60	Shufu County 15; Yingjisha County 25; Shache County 10; Yecheng County 10
Hotan	30	30	Pishan County 15; Cele County 15
Ili	30	30	Yining County 10; Chabuchaer County 10; Gongliu County 10
Hami	5	5	Yizhou District 5
Kizilsu		5	Akto County 5
Total	205	205	

Appendix Table 1

2025 Quality and Safety Monitoring Schedule for Edible Forest Products and Soils in Their Production Areas

Apples | Monitoring time: Early-season: July; mid-season: September; late-season: October

Prefecture / city	Product batches	Soil batches	County/site allocation for each sample type
Aksu	65	65	Hongqipo Farm 30; Aksu City 20; Wensu County 15
Ili	30	30	Gongliu County 10; Yining County 10; Chabuchaer County 10
Kashgar	25	25	Yecheng County 5; Zepu County 10; Shule County 10
Total	120	120	

Fragrant pears | Monitoring time: September

Prefecture / city	Product batches	Soil batches	County/site allocation for each sample type
Bayingolin	60	60	Korla City 50; Luntai County 5; Yuli County 5
Aksu	30	30	Aksu City 10; Kuqa City 15; Awati County 5
Total	90	90	

Appendix Table 1

2025 Quality and Safety Monitoring Schedule for Edible Forest Products and Soils in Their Production Areas

Grapes | Monitoring time: July in Turpan; August elsewhere

Prefecture / city	Product batches	Soil batches	County/site allocation for each sample type
Turpan	55	55	Gaochang District 25; Shanshan County 30
Hami	5	5	Yizhou District 5
Bayingolin	5	5	Heshuo County 5
Kashgar	5	5	Kashgar City 5
Hotan	10	10	Yutian County 5; Moyu County 5
Changji	5	5	Changji City 5
Kizilsu	5	5	Artux City 5
Total	90	90	

Sea buckthorn | Monitoring time: September

Prefecture / city	Product batches	Soil batches	County/site allocation for each sample type
Altay	15	15	Burqin County 5; Habahe County 5; Qinghe County 5
Aksu	15	15	Wensu County 5; Wushi County 10
Kizilsu	5	5	Aheqi County 5
Total	35	35	

Appendix Table 1

2025 Quality and Safety Monitoring Schedule for Edible Forest Products and Soils in Their Production Areas

Almonds | Monitoring time: July

Prefecture / city	Product batches	Soil batches	County/site allocation for each sample type
Kashgar	30	30	Shache County 30
Total	30	30	

Goji berries | Monitoring time: July

Prefecture / city	Product batches	Soil batches	County/site allocation for each sample type
Bortala	10	10	Jinghe County 10
Tacheng	2	2	Wusu City 2
Bayingolin	3	3	Yuli County 3
Changji	5	5	Manas County 5
Total	20	20	

Peaches | Monitoring time: July

Prefecture / city	Product batches	Soil batches	County/site allocation for each sample type
Kashgar	20	20	Shule County 10; Yingjisha County 5; Shache County 5
Hotan	5	5	Hotan County 3; Moyu County 2
Total	25	25	

Appendix Table 1

2025 Quality and Safety Monitoring Schedule for Edible Forest Products and Soils in Their Production Areas

Pomegranates | Monitoring time: October

Prefecture / city	Product batches	Soil batches	County/site allocation for each sample type
Kashgar	10	10	Kashgar City 5; Jiashi County 5
Hotan	10	10	Pishan County 5; Cele County 5
Total	20	20	

Xinmei plums | Monitoring time: August

Prefecture / city	Product batches	Soil batches	County/site allocation for each sample type
Kashgar	60	60	Jiashi County 40; Yuepuhu County 10; Shache County 10
Ili	10	10	Yining County 5; Chabuchaer County 5
Total	70	70	

Cistanche | Monitoring time: October

Prefecture / city	Product batches	Soil batches	County/site allocation for each sample type
Hotan	25	25	Yutian County 15; Cele County 5; Hotan County 5
Bayingolin	5	5	Qiemo County 5
Total	30	30	

Grand total: 2,200 batches (1,100 product batches and 1,100 production-area soil batches).

Appendix Table 2

Sample Quantities

Product category	Initial-test sample	Retest sample
Walnuts	1,000 g	1,000 g
Red jujubes	1,000 g	1,000 g
Apricots	1,000 g	1,000 g
Apples	2,000 g (>5 fruits)	2,000 g (>5 fruits)
Fragrant pears	1,000 g	1,000 g
Grapes	1,000 g	1,000 g
Sea buckthorn	1,000 g	1,000 g
Almonds	1,000 g	1,000 g
Goji berries	1,000 g	1,000 g
Peaches	1,000 g	1,000 g
Pomegranates	2,000 g (>5 fruits)	2,000 g (>5 fruits)
Xinmei plums	1,000 g	1,000 g
Cistanche	1,000 g (>3 stems)	1,000 g (>3 stems)
Soil	1,000 g	1,000 g

Note: The sample quantities refer to the amount of edible material required for each analytical method after removal of the peel and core from the forest-fruit products.

Appendix Table 3

2025 Quality and Safety Monitoring Items and Applicable Standards for Edible Forest Products

Walnuts

Pesticide residues: 26 items

1. difenoconazole	10. chlorpyrifos	19. chlorobenzilate
2. pendimethalin	11. diazinon	20. cypermethrin and beta-cypermethrin
3. fenpropathrin	12. phosalone	21. cyhalothrin and lambda-cyhalothrin
4. methoxychlor	13. dicofol	22. phosmet
5. fenbuconazole	14. tetrachlorvinphos	23. etoxazole
6. chloroneb	15. methidathion	24. deltamethrin
7. boscalid	16. mevinphos	25. endosulfan
8. fluorodifen	17. trifloxystrobin	26. chlordane
9. bifenthrin	18. tebuconazole	

Assessment standard: GB 2763-2021
Analytical standards: GB 23200.113

Heavy metals - 1 item: lead
Assessment standard: GB 2762-2022
Analytical standards: GB 5009.12

Appendix Table 3

2025 Quality and Safety Monitoring Items and Applicable Standards for Edible Forest Products

Red jujubes

Pesticide residues: 52 items

1. aldrin	19. myclobutanil	37. carbofuran
2. fenthion	20. omethoate	38. dimethoate
3. propiconazole	21. chloroneb	39. triadimefon
4. fonofos	22. dicofol	40. fenitrothion
5. dieldrin	23. tetrachlorvinphos	41. isocarbophos
6. dichlorvos	24. methidathion	42. phosfolan
7. parathion	25. mevinphos	43. cypermethrin and beta-cypermethrin
8. phosalone	26. trifloxystrobin	44. cyfluthrin and beta-cyfluthrin
9. fludioxonil	27. chlorobenzilate	45. fenvalerate and esfenvalerate
10. methamidophos	28. endrin	46. deltamethrin
11. parathion-methyl	29. coumaphos	47. abamectin
12. isofenphos-methyl	30. sulfotep	48. acetamiprid
13. fenpropathrin	31. permethrin	49. carbendazim
14. methoxychlor	32. isazofos	50. novaluron
15. monocrotophos	33. malathion	51. phoxim
16. pirimicarb	34. kresoxim-methyl	52. fenamiphos
17. boscalid	35. cyprodinil	
18. fluorodifen	36. ethoprophos	

Assessment standard: GB 2763-2021
Analytical standards: GB 23200.121; GB 23200.113

Heavy metals - 1 item: lead and cadmium
Assessment standard: GB 2762-2022
Analytical standards: GB 5009.12; GB 5009.15

Appendix Table 3

2025 Quality and Safety Monitoring Items and Applicable Standards for Edible Forest Products

Apricots

Pesticide residues: 53 items

1. aldrin	19. boscalid	37. dimethoate
2. fenthion	20. fluorodifen	38. pyrimethanil
3. fonofos	21. myclobutanil	39. tebuconazole
4. dieldrin	22. chloroneb	40. fenitrothion
5. dichlorvos	23. dicofol	41. isocarbophos
6. chlorpyrifos	24. tetrachlorvinphos	42. cypermethrin and beta-cypermethrin
7. parathion	25. methidathion	43. cyhalothrin and lambda-cyhalothrin
8. phosalone	26. mevinphos	44. phosfolan
9. fludioxonil	27. trifloxystrobin	45. ethoprophos
10. methamidophos	28. omethoate	46. fenvalerate and esfenvalerate
11. parathion-methyl	29. chlorobenzilate	47. deltamethrin
12. isofenphos-methyl	30. endrin	48. phosmet
13. fenpropathrin	31. coumaphos	49. abamectin
14. methoxychlor	32. sulfotep	50. acetamiprid
15. fenbuconazole	33. permethrin	51. novaluron
16. monocrotophos	34. isazofos	52. spirodiclofen
17. pirimicarb	35. malathion	53. phoxim
18. carbofuran	36. cyprodinil	

Assessment standard: GB 2763-2021
Analytical standards: GB 23200.121; GB 23200.113

Heavy metals - 1 item: lead and cadmium
Assessment standard: GB 2762-2022
Analytical standards: GB 5009.12; GB 5009.15

Appendix Table 3

2025 Quality and Safety Monitoring Items and Applicable Standards for Edible Forest Products

Apples

Pesticide residues: 82 items

1. chlorothalonil	29. fluorodifen	57. tetradifon
2. aldrin	30. myclobutanil	58. bromopropylate
3. fenthion	31. bifenthrin	59. triadimenol
4. difenoconazole	32. chloroneb	60. diniconazole
5. propiconazole	33. dicofol	61. fenitrothion
6. profenofos	34. tetrachlorvinphos	62. fenarimol
7. pyridaben	35. methidathion	63. Cypermethrin and beta-cypermethrin
8. fonofos	36. mevinphos	64. cyfluthrin and beta-cyfluthrin
9. dieldrin	37. trifloxystrobin	65. fenvalerate and esfenvalerate
10. dichlorvos	38. omethoate	66. phosfolan
11. penconazole	39. chlorobenzilate	67. triazophos
12. chlorpyrifos	40. endrin	68. isocarbophos
13. parathion	41. iprodione	69. deltamethrin
14. diazinon	42. imazalil	70. phosmet
15. phosalone	43. coumaphos	71. oxyfluorfen
16. epoxiconazole	44. sulfotep	72. abamectin
17. flucythrinate	45. permethrin	73. carbendazim
18. fludioxonil	46. isazofos	74. novaluron
19. hexaconazole	47. malathion	75. acetamiprid
20. methamidophos	48. kresoxim-methyl	76. thiophanate-methyl
21. parathion-methyl	49. cyprodinil	77. cadusafos
22. isofenphos-methyl	50. pyrimethanil	78. spirodiclofen
23. fenpropathrin	51. ethoprophos	79. phoxim
24. methoxychlor	52. carbofuran	80. fenamiphos
25. fenbuconazole	53. dimethoate	81. imidacloprid
26. monocrotophos	54. triadimefon	82. pyraclostrobin
27. pirimicarb	55. tebuconazole	
28. boscalid	56. etoxazole	

Assessment standard: GB 2763-2021
Analytical standards: NY/T 761; GB 23200.121; GB 23200.113

Heavy metals - 2 items: lead and cadmium
Assessment standard: GB 2762-2022
Analytical standards: GB 5009.12; GB 5009.15

Appendix Table 3

2025 Quality and Safety Monitoring Items and Applicable Standards for Edible Forest Products

Fragrant pears

Pesticide residues: 77 items

1. chlorothalonil	27. myclobutanil	53. bromopropylate
2. aldrin	28. difenoconazole	54. diniconazole
3. fenthion	29. penconazole	55. fenitrothion
4. fonofos	30. chloroneb	56. isocarbophos
5. dieldrin	31. dicofol	57. cypermethrin and beta-cypermethrin
6. dichlorvos	32. tetrachlorvinphos	58. deltamethrin
7. chlorpyrifos	33. methidathion	59. cyhalothrin and lambda-cyhalothrin
8. parathion	34. mevinphos	60. cyfluthrin and beta-cyfluthrin
9. diazinon	35. trifloxystrobin	61. phosfolan
10. phosalone	36. omethoate	62. fenarimol
11. flucythrinate	37. chlorobenzilate	63. fenvalerate and esfenvalerate
12. fludioxonil	38. endrin	64. phosmet
13. hexaconazole	39. iprodione	65. abamectin
14. methamidophos	40. imazalil	66. thiophanate-methyl
15. parathion-methyl	41. coumaphos	67. carbendazim
16. isofenphos-methyl	42. sulfotep	68. novaluron
17. fenpropathrin	43. permethrin	69. acetamiprid
18. methoxychlor	44. isazofos	70. cadusafos
19. fenbuconazole	45. malathion	71. spirodiclofen
20. monocrotophos	46. kresoxim-methyl	72. phoxim
21. pirimicarb	47. cyprodinil	73. fenpyroximate
22. carbofuran	48. pyrimethanil	74. fenamiphos
23. dimethoate	49. ethoprophos	75. imidacloprid
24. bifenthrin	50. triadimefon	76. pyraclostrobin
25. phosphamidon	51. tebuconazole	77. diflubenzuron
26. fluorodifen	52. etoxazole	

Assessment standard: GB 2763-2021
Analytical standards: NY/T 761; GB 23200.121; GB 23200.113

Heavy metals - 1 item: lead and cadmium
Assessment standard: GB 2762-2022
Analytical standards: GB 5009.12; GB 5009.15

Appendix Table 3

2025 Quality and Safety Monitoring Items and Applicable Standards for Edible Forest Products

Grapes

Pesticide residues: 76 items

1. chlorothalonil	27. boscalid	53. isocarbophos
2. aldrin	28. fluorodifen	54. fenarimol
3. fenthion	29. myclobutanil	55. tebuconazole
4. difenoconazole	30. chloroneb	56. etoxazole
5. fonofos	31. permethrin	57. diniconazole
6. dieldrin	32. isazofos	58. bromopropylate
7. dichlorvos	33. malathion	59. cypermethrin and beta-cypermethrin
8. chlorpyrifos	34. kresoxim-methyl	60. cyfluthrin and beta-cyfluthrin
9. parathion	35. cyprodinil	61. fenvalerate and esfenvalerate
10. epoxiconazole	36. pyrimethanil	62. deltamethrin
11. procymidone	37. ethoprophos	63. phosmet
12. fludioxonil	38. dicofol	64. abamectin
13. hexaconazole	39. triadimefon	65. acetamiprid
14. methamidophos	40. tetrachlorvinphos	66. carbendazim
15. parathion-methyl	41. mevinphos	67. thiophanate-methyl
16. isofenphos-methyl	42. trifloxystrobin	68. cadusafos
17. fenpropathrin	43. penconazole	69. spirodiclofen
18. methoxychlor	44. omethoate	70. demeton
19. fenbuconazole	45. chlorobenzilate	71. phoxim
20. monocrotophos	46. endrin	72. fenpyroximate
21. pirimicarb	47. iprodione	73. fenamiphos
22. carbofuran	48. imazalil	74. imidacloprid
23. quinoxyfen	49. coumaphos	75. pyraclostrobin
24. dimethoate	50. sulfotep	76. heptenophos
25. bifenthrin	51. triadimenol	
26. phosphamidon	52. fenitrothion	

Assessment standard: GB 2763-2021
Analytical standards: NY/T 761; GB 23200.121; GB 23200.113

Heavy metals - 1 item: lead and cadmium
Assessment standard: GB 2762-2022
Analytical standards: GB 5009.12; GB 5009.15

Appendix Table 3

2025 Quality and Safety Monitoring Items and Applicable Standards for Edible Forest Products

Sea buckthorn

Pesticide residues: 43 items

1. fenthion	16. permethrin	31. aldrin
2. dichlorvos	17. isazofos	32. dieldrin
3. fonofos	18. cyprodinil	33. fenitrothion
4. parathion	19. pyrimethanil	34. cypermethrin and beta-cypermethrin
5. methamidophos	20. ethoprophos	35. monocrotophos
6. parathion-methyl	21. dicofol	36. fenvalerate and esfenvalerate
7. isofenphos-methyl	22. boscalid	37. isocarbophos
8. fenpropathrin	23. fluorodifen	38. endrin
9. methoxychlor	24. tetrachlorvinphos	39. acetamiprid
10. pirimicarb	25. methidathion	40. carbendazim
11. carbofuran	26. mevinphos	41. cadusafos
12. dimethoate	27. omethoate	42. imidacloprid
13. phosphamidon	28. chlorobenzilate	43. phoxim
14. phosfolan	29. coumaphos	
15. chloroneb	30. sulfotep	

Assessment standard: GB 2763-2021
Analytical standards: GB 23200.121; GB 23200.113

Heavy metals - 1 item: lead and cadmium
Assessment standard: GB 2762-2022
Analytical standards: GB 5009.12; GB 5009.15

Appendix Table 3

2025 Quality and Safety Monitoring Items and Applicable Standards for Edible Forest Products

Almonds

Pesticide residues: 22 items

1. difenoconazole	9. mevinphos	17. cyhalothrin and lambda-cyhalothrin
2. pendimethalin	10. trifloxystrobin	18. endosulfan
3. fenpropathrin	11. methoxychlor	19. phosmet
4. boscalid	12. fenbuconazole	20. etoxazole
5. fluorodifen	13. chloroneb	21. chlorobenzilate
6. dicofol	14. bifenthrin	22. chlordane
7. tetrachlorvinphos	15. tebuconazole	
8. methidathion	16. cypermethrin and beta-cypermethrin	

Assessment standard: GB 2763-2021
Analytical standards: GB 23200.113

Heavy metals - 1 item: lead
Assessment standard: GB 2762-2022
Analytical standards: GB 5009.12

Appendix Table 3

2025 Quality and Safety Monitoring Items and Applicable Standards for Edible Forest Products

Goji berries

Pesticide residues: 48 items

1. chlorothalonil	17. permethrin	33. endrin
2. fenthion	18. isazofos	34. pyridaben
3. dichlorvos	19. cyprodinil	35. chlorpyrifos
4. fonofos	20. pyrimethanil	36. hexaconazole
5. parathion	21. ethoprophos	37. etoxazole
6. methamidophos	22. dicofol	38. cypermethrin and beta-cypermethrin
7. parathion-methyl	23. boscalid	39. monocrotophos
8. isofenphos-methyl	24. fluorodifen	40. fenvalerate and esfenvalerate
9. fenpropathrin	25. tetrachlorvinphos	41. fenitrothion
10. methoxychlor	26. mevinphos	42. isocarbophos
11. pirimicarb	27. omethoate	43. abamectin
12. carbofuran	28. chlorobenzilate	44. acetamiprid
13. dimethoate	29. coumaphos	45. carbendazim
14. phosphamidon	30. sulfotep	46. cadusafos
15. phosfolan	31. aldrin	47. phoxim
16. chloroneb	32. dieldrin	48. fenpyroximate

Assessment standard: GB 2763-2021
Analytical standards: NY/T 761; GB 23200.121; GB 23200.113

Heavy metals - 1 item: lead and cadmium
Assessment standard: GB 2762-2022
Analytical standards: GB 5009.12; GB 5009.15

Appendix Table 3

2025 Quality and Safety Monitoring Items and Applicable Standards for Edible Forest Products

Peaches

Pesticide residues: 63 items

1. chlorothalonil	22. fluorodifen	43. iprodione
2. aldrin	23. myclobutanil	44. coumaphos
3. fenthion	24. pirimicarb	45. sulfotep
4. difenoconazole	25. carbofuran	46. fenitrothion
5. propiconazole	26. dimethoate	47. isocarbophos
6. fonofos	27. chloroneb	48. tebuconazole
7. dieldrin	28. permethrin	49. phosfolan
8. dichlorvos	29. isazofos	50. fenarimol
9. chlorpyrifos	30. malathion	51. cypermethrin and beta-cypermethrin
10. parathion	31. cyprodinil	52. cyhalothrin and lambda-cyhalothrin
11. diazinon	32. pyrimethanil	53. cyfluthrin and beta-cyfluthrin
12. phosalone	33. ethoprophos	54. fenvalerate and esfenvalerate
13. fludioxonil	34. dicofol	55. deltamethrin
14. methamidophos	35. tetrachlorvinphos	56. phosmet
15. parathion-methyl	36. methidathion	57. abamectin
16. isofenphos-methyl	37. mevinphos	58. acetamiprid
17. fenpropathrin	38. trifloxystrobin	59. carbendazim
18. methoxychlor	39. penconazole	60. novaluron
19. fenbuconazole	40. omethoate	61. spirodiclofen
20. monocrotophos	41. chlorobenzilate	62. phoxim
21. boscalid	42. endrin	63. fenamiphos

Assessment standard: GB 2763-2021
Analytical standards: NY/T 761; GB 23200.121; GB 23200.113

Heavy metals - 1 item: lead and cadmium
Assessment standard: GB 2762-2022
Analytical standards: GB 5009.12; GB 5009.15

Appendix Table 3

2025 Quality and Safety Monitoring Items and Applicable Standards for Edible Forest Products

Pomegranates

Pesticide residues: 40 items

1. fenthion	15. chloroneb	29. sulfotep
2. dichlorvos	16. permethrin	30. aldrin
3. fonofos	17. isazofos	31. endrin
4. parathion	18. ethoprophos	32. difenoconazole
5. methamidophos	19. dicofol	33. fludioxonil
6. parathion-methyl	20. tetrachlorvinphos	34. fenvalerate and esfenvalerate
7. isofenphos-methyl	21. fenitrothion	35. dieldrin
8. fenpropathrin	22. methidathion	36. acetamiprid
9. methoxychlor	23. isocarbophos	37. cadusafos
10. monocrotophos	24. mevinphos	38. imidacloprid
11. carbofuran	25. fluorodifen	39. demeton
12. dimethoate	26. omethoate	40. phoxim
13. phosphamidon	27. chlorobenzilate	
14. phosfolan	28. coumaphos	

Assessment standard: GB 2763-2021
Analytical standards: GB 23200.121; GB 23200.113

Heavy metals - 1 item: lead and cadmium
Assessment standard: GB 2762-2022
Analytical standards: GB 5009.12; GB 5009.15

Appendix Table 3

2025 Quality and Safety Monitoring Items and Applicable Standards for Edible Forest Products

Xinmei plums

Pesticide residues: 46 items

1. aldrin	17. phosfolan	33. chlorobenzilate
2. fenthion	18. boscalid	34. isocarbophos
3. fonofos	19. fluorodifen	35. coumaphos
4. dieldrin	20. myclobutanil	36. cypermethrin and beta-cypermethrin
5. dichlorvos	21. chloroneb	37. fenvalerate and esfenvalerate
6. parathion	22. permethrin	38. endrin
7. phosalone	23. isazofos	39. sulfotep
8. fludioxonil	24. cyprodinil	40. acetamiprid
9. methamidophos	25. ethoprophos	41. novaluron
10. parathion-methyl	26. dicofol	42. phoxim
11. isofenphos-methyl	27. tetrachlorvinphos	43. demeton
12. fenpropathrin	28. methidathion	44. fenpyroximate
13. methoxychlor	29. mevinphos	45. fenamiphos
14. monocrotophos	30. trifloxystrobin	46. heptenophos
15. carbofuran	31. omethoate	
16. dimethoate	32. fenitrothion	

Assessment standard: GB 2763-2021
Analytical standards: GB 23200.121; GB 23200.113

Heavy metals - 1 item: lead and cadmium
Assessment standard: GB 2762-2022
Analytical standards: GB 5009.12; GB 5009.15

Appendix Table 3

2025 Quality and Safety Monitoring Items and Applicable Standards for Edible Forest Products

Cistanche

Pesticide residues: 13 items

1. isofenphos-methyl	6. dicofol	11. mevinphos
2. methoxychlor	7. tetrachlorvinphos	12. heptenophos
3. carbofuran	8. methidathion	13. chlorsulfuron
4. dimethoate	9. chlorobenzilate	
5. chloroneb	10. fluorodifen	

Assessment standard: GB 2763-2021
Analytical standards: GB 23200.121; GB 23200.113

Appendix Table 4

2025 Quality and Safety Monitoring Items and Applicable Standards for Production-Area Soils

Sample type: soils from edible forest-product production areas.
Assessment standard: GB 15618-2018.
Values are maximum concentrations (mg/kg); concentrations shall not exceed the applicable value.

No.	Analyte	pH <=5.5	5.5<pH <=6.5	6.5<pH <=7.5	pH >7.5	Analytical standard
1	Cadmium	0.3	0.3	0.3	0.6	GB/T 17141
2	Mercury	1.3	1.8	2.4	3.4	NY/T 1121.10
3	Arsenic	40	40	30	25	NY/T 1121.11
4	Lead	70	90	120	170	GB/T 17141
5	Chromium	150	150	200	250	HJ 491
6	Copper	150	150	200	200	HJ 491
7	Nickel	60	70	100	190	HJ 491
8	Zinc	200	200	250	250	HJ 491
9	Total HCH	0.10	0.10	0.10	0.10	GB/T 14550
10	Total DDT	0.10	0.10	0.10	0.10	GB/T 14550
11	Benzo[a]pyrene	0.55	0.55	0.55	0.55	HJ 805

Notes:
Total HCH is the sum of alpha-HCH, beta-HCH, gamma-HCH and delta-HCH.
Total DDT is the sum of p,p'-DDE, p,p'-DDD, o,p'-DDT and p,p'-DDT.
