# Supplementary material for: Multi-Pesticide Residue Occurrence and Age-Specific Dietary Exposure Risk Assessment of Apples from Major Production Areas in Xinjiang, China
Source: Foods. 2026 Sep 29;15(19):3491. doi: 10.3390/foods15193491 (PMC13665483; doi:10.3390/foods15193491)
Supplement: Supplementary file 1 [file foods-15-03491-s001.zip › Monitoring notice chinese.pdf]

新疆维吾尔自治区林业和草原局

新林办函〔2025〕198号

关于开展2025年自治区食用林产品及其产地土壤质量安全监测工作的通知

各地、州、市林业和草原局：

根据《国家林业和草原局办公室关于印发2023-2025年省级食用林产品质量安全监测工作计划的通知（办科字〔2023〕68号）要求，结合新疆食用林产品质量安全监测实际，制定《2025年自治区食用林产品及其产地土壤质量安全监测方案》（详见附件，以下简称《方案》），现将《方案》印发给你们，并就有关事项通知如下：

一、配合做好监测工作。各地林业和草原主管部门要严格按照《方案》要求，主动与技术支持单位沟通，确保监测工作有序推进，按时保质保量完成。要督促各受检单位切实履行生产经营者的产品质量安全主体责任，积极配合抽样和监测工作。

二、规范监测工作程序。各地林业和草原主管部门要加强对监测工作的统筹，规范从业人员行为，规范选择抽样区域，规范取样，规范运输，确保监测工作的科学性、公正性、真实性、准确性。技术支持单位发现问题，应及时反馈至各地林业和草原主管部门，并将有关情况上报自治区林业和草原局。

三、提高自身监测水平。鼓励各级林果业主管部门在自治区林业和草原局开展林果质量安全监测的基础上，结合当地林果业

发展实际，依托有资质的科研检测机构，自行组织林果产品质量安全监测，提升当地食用林产品质量安全监测指标、监测树种、监测区域覆盖面。

请各单位结合自身实际认真抓好贯彻落实，并于4月25日前反馈工作人员（单位、姓名、职务职称、联系电话、电子邮箱）至技术支持单位自治区林业科学院林草资源研究中心。

联 系 人：杨 璐

联系电话：0991-4649261　18899161201

电子邮箱：lycszx_office@163.com

通讯地址：乌鲁木齐市水磨沟区安居南路191号自治区林业科学院

邮政编码：830000

附件：2025年自治区食用林产品及其产地土壤质量安全监测方案

自治区林业和草原局

2025年4月22日

抄送：自治区林业科学院。

内发：余副局长，办公室、科技处。

新疆维吾尔自治区林业和草原局办公室　　2025年4月22日印发

附件

2025年自治区食用林产品及其产地土壤质量安全监测方案

一、监测产品

监测的食用林产品包括：核桃、红枣、杏、苹果、香梨、葡萄、巴旦木、枸杞、桃、石榴、新梅、沙棘共12大类林果产品的鲜果，以及林源药材1种：肉苁蓉。

监测的食用林产品产地土壤包括：核桃产地土壤、红枣产地土壤、杏产地土壤、苹果产地土壤、香梨产地土壤、葡萄产地土壤、巴旦木产地土壤、枸杞产地土壤、桃产地土壤、石榴产地土壤、新梅产地土壤、沙棘产地土壤、肉苁蓉产地土壤。

二、监测地区和数量

食用林产品生产重点县（市、区）管辖范围内的规模化种植基地和个体果园，每个县（市、区）抽检地点不少于3个村，具体监测地区、监测数量及承检机构见附表1。

2025年共监测2200批次，其中：（1）拟监测食用林产品1100批次，其中核桃185批次、红枣180批次、杏205批次、苹果120批次、香梨90批次、葡萄90批次、巴旦木30批次、枸杞20批次、桃25批次、石榴20批次、新梅70批次、沙棘35批次、肉苁蓉30批次。（2）拟监测食用林产品产地土壤1100

批次，其中核桃产地土壤185批次、红枣产地土壤180批次、杏产地土壤205批次、苹果产地土壤120批次、香梨产地土壤90批次、葡萄产地土壤90批次、巴旦木产地土壤30批次、枸杞产地土壤20批次、桃产地土壤25批次、石榴产地土壤20批次、新梅产地土壤70批次、沙棘产地土壤35批次、肉苁蓉产地土壤30批次（具体监测地区、监测数量见附表1）。

三、抽样

（一）抽样区域

在规模化种植基地、合作社、个体种植园、有机种植园抽样。监测抽样地点应具有代表性，要求在主产区抽样，每一个采样点或采样区域原则上抽取不超过5个样品（要求种植面积50亩以上），食用林产品及其产地土壤的抽样应具有对应性，监测样品应能反映当地被监测的食用林产品及其产地土壤的质量安全水平。

（二）抽样要求

由被监测市、县（区）林草主管部门根据当地林果产品生产情况提供有代表性的生产基地名单，承检机构从中随机选取，并由当地林草行政主管部门抽样人员独立抽样并送至承检机构。承检机构随机抽取被监测市、县（区）与当地采样人员协同抽样。抽样地点应具有代表性，能反映当地被监测的林果产品质量安全水平。凡被确定的抽样基地不得拒绝抽检，否则其产品按不合格处理。

为便于今后追溯问题产品来源，抽样人员必须严格按照《食用林产品质量安全监测抽样单》内容填写，并向被检测单位或个人索要盖章和签名。

（三）抽样环节

生产基地环节—各监测县（市）抽检产品的主要生产基地。

（四）抽样方法

食用林产品参照《经济林产品质量安全监测技术规程》（LY/T 2800-2017）规定执行。(1)鲜样抽检:核桃、红枣、杏、苹果、香梨、葡萄、巴旦木、枸杞、桃、石榴、新梅、沙棘、肉苁蓉应从种植基地成熟期的产品中抽取;抽检的每个林果产品应分为初检样品和复检样品2份（产品的具体抽样数量见附表2），采用干净无污染的容器或塑料袋装样。包装后的林果产品用封条进行封样，抽样人员与被监测单位代表在封条上签字。

土壤采集按《土壤环境监测技术规范》（HJ/T 166-2004）规定执行，采集0-40cm土壤，每个采样区的土壤样品为一个混合样，混合样采集点数不少于5个，混合后的土壤样品重量不少于1kg，采用干净无污染的容器或塑料袋装样，多个样点土样充分混匀后包装，用封条进行封样，抽样人员与被监测单位代表在封条上签字。

（五）抽样基数

以按照基地生产管理要求生产的同一批次采收的产品为抽样基数，原则上抽样基数不少于200公斤。

（六）抽样数量

每类产品具体抽样数量详见附表2。

（七）样品运输与保藏

食用林产品的初检样品和复检样品在运输、存储过程中应防止污染和变质，不得受潮、雨淋和曝晒。样品到达承检机构时，承检机构应对样品状态进行验收，包括封条是否完好，样品包装是否损坏等，如样品已经损坏、变质，应重新抽样。验收合格后，样品应及时处理，初检样品按对应的检测方法标准要求进行制样和测定，复检样品原样冷冻存储。产地土壤样品在运输过程中应包装牢固严防样品的损失，土壤样品到达承检机构时，承检机构应对样品状态进行验收，包括封条是否完好，样品包装是否损坏等，如样品包装已经损坏应重新抽样，土壤样品按对应的检测方法标准要求进行制样和测定。

（八）抽样人员

各级林草主管部门安排抽样人员。抽样人员必须持有效证件、抽查文件、记录本、抽样单和调查表等。

四、监测项目和检测依据

监测项目及检测依据见附表3和附表4。

五、判定依据和原则

依据《食品安全国家标准 食品中污染物限量》（GB 2762-2022）和《食品安全国家标准 食品中农药最大残留限量》（GB 2763-2021）中的限量值进行判定。监测项目全部合格者，判定为“该监测抽检

××产品检测项目符合××标准限量要求”；监测项目中任一项或一项以上不合格，按标准中规定的复检要求检验后仍不符合检验规定的，判定为“该监测抽检××产品检测项目××不符合××标准限量要求，其他检测项目符合××标准限量要求”。

产地土壤监测项目依据《土壤环境质量农用地土壤污染风险管控标准（试行）》（GB 15618-2018）中的风险筛选值和风险管制值进行判定。具体判定如下：检验项目全部合格，判定为“该产地土壤检测项目符合 GB 15618-2018 标准风险筛选值要求”；检测项目中任一项或一项以上不合格，按标准中规定的复检要求对该项进行检验后仍不符合判定依据规定的，判定为“该产地土壤检测项目不符合 GB 15618-2018 标准风险筛选值要求，其他检测项目符合 GB 15618-2018 标准风险筛选值要求”。

六、数据报送

承检机构在样品测试完毕后，及时进行数据汇总，填写数据信息汇总表，汇总数据格式应按相应的检测标准中规定的要求对保留位数进行数字修约，如检测标准中未规定检测结果的数字保留位数，统一检测结果数据保留 3 位有效数字，对未检出的项目，承检机构应提供相应的检测方法的检出限。同时承检机构要对监测结果进行综合评价和原因分析，以自治区食用林产品质量年度报告形式报送上级主管部门。

七、时间安排

根据被监测食用林产品成熟时间安排监测抽样和检测工作，

一般情况下应在食用林产品成熟期当月完成抽样、检测工作。具体如下：

2025 年 6 月 30 日前，完成杏及其产地土壤的抽样和检测工作；

2025 年 7 月 30 日前，完成桃、枸杞、巴旦木及其产地土壤的抽样和检测工作；

2025 年 8 月 30 日前，完成中早熟葡萄、新梅及其产地土壤的抽样和检测工作；

2025 年 9 月 30 日前，完成晚熟葡萄、香梨、核桃、沙棘及其产地土壤的抽样和检测工作；

2025 年 10 月 30 日前，完成苹果、石榴、红枣、肉苁蓉及其产地土壤的抽样和检测工作；

2025 年 11 月 30 日，检测机构完成所有材料收集汇总，将监测工作相关材料报送自治区林草局。

八、工作要求

（一）监测工作应按照《食用林产品质量安全监测工作规范(2020 年版)》进行。

（二）监测工作应保持科学性、代表性和真实性。

（三）参与抽样工作的各级林草主管部门、抽样单位、承检机构及其工作人员要依法进行抽样、检验和结果上报，不得随意更改抽样地点和样品信息，不得瞒报、谎报、漏报检测数据，确保结果的真实、客观和准确。

（四）未经自治区林业和草原局许可，任何单位和个人不得公布监测结果。

（五）抽样完成后要及时将样品送回检测室，在运输过程中要避免样品受到污染和变质。

附件：1. 2025 年食用林产品及其产地土壤质量安全监测表
2. 抽样数量
3. 2025 年食用林产品质量安全监测项目及依据标准
4. 2025 年产地土壤质量安全监测项目及依据标准

附表 1

2025 年食用林产品及其产地土壤质量安全监测表

监测品种	监测时间	抽样地区及监测批次	
核桃	9 月	阿克苏地区（75 批次）	阿克苏市（15）、温宿县（30）、库车市（10）、新和县（10）、乌什县（10）
		喀什地区（60 批次）	泽普县（15）、莎车县（15）、叶城县（20）、麦盖提县（5）、巴楚县（5）
		和田地区（50 批次）	和田县（10）、墨玉县（15）、皮山县（5）、洛浦县（5）、于田县（5）、民丰县（10）
		**合计：185 批次**	
核桃产地土壤	9 月	阿克苏地区（75 批次）	阿克苏市（15）、温宿县（30）、库车市（10）、新和县（10）、乌什县（10）
		喀什地区（60 批次）	泽普县（15）、莎车县（15）、叶城县（20）、麦盖提县（5）、巴楚县（5）
		和田地区（50 批次）	和田县（10）、墨玉县（15）、皮山县（5）、洛浦县（5）、于田县（5）、民丰县（10）
		**合计：185 批次**	

监测品种	监测时间	抽样地区及监测批次	
红枣	10月	哈密市（5 批次）	伊州区（5）
		巴州（30 批次）	若羌县（20）、且末县（10）
		阿克苏地区（30 批次）	阿克苏市（15）、温宿县（15）
		喀什地区（60 批次）	泽普县（10）、麦盖提县（30）、岳普湖县（10）、巴楚县（10）
		和田地区（55 批次）	和田县（15）、洛浦县（15）、策勒县（15）、墨玉县（10）
		**合计：180 批次**	
红枣产地土壤	10月	哈密市（5 批次）	伊州区（5）
		巴州（30 批次）	若羌县（20）、且末县（10）
		阿克苏地区（30 批次）	阿克苏市（15）、温宿县（15）
		喀什地区（60 批次）	泽普县（10）、麦盖提县（30）、岳普湖县（10）、巴楚县（10）
		和田地区（55 批次）	和田县（15）、洛浦县（15）、策勒县（15）、墨玉县（10）
		**合计：180 批次**	
杏	吐鲁番地区 5 月；其他地区 6 月、7 月	吐鲁番市（30 批次）	高昌区（10）、托克逊县（20）
		巴州（25 批次）	轮台县（20）、和硕县（5）
		阿克苏地区（20 批次）	库车市（15）、温宿县（5）
		喀什地区（60 批次）	疏附县（15）、英吉沙县（25）、莎车县（10）、叶城县（10）

监测品种	监测时间	抽样地区及监测批次	
		和田地区（30 批次）	皮山县（15）、策勒县（15）
		伊犁州（30 批次）	伊宁县（10）、察布查尔县（10）、巩留县（10）
		哈密市（5 批次）	伊州区（5）
		克州	阿克陶县（5）
		**合计：205 批次**	
杏产地土壤	吐鲁番地区 5 月；其他地区 6 月、7 月	吐鲁番市（30 批次）	高昌区（10）、托克逊县（20）
		巴州（25 批次）	轮台县（20）、和硕县（5）
		阿克苏地区（20 批次）	库车市（15）、温宿县（5）
		喀什地区（60 批次）	疏附县（15）、英吉沙县（25）、莎车县（10）、叶城县（10）
		和田地区（30 批次）	皮山县（15）、策勒县（15）
		伊犁州（30 批次）	伊宁县（10）、察布查尔县（10）、巩留县（10）
		哈密市（5 批次）	伊州区（5）
		克州（5 批次）	阿克陶县（5）
		**合计：205 批次**	
苹果	早熟品种 7 月、中熟品种 9 月、晚熟品	阿克苏地区（65 批次）	红旗坡农场（30）、阿克苏市（20）、温宿县（15）
		伊犁州（30 批次）	巩留县（10）、伊宁县（10）、察布查尔县（10）
		喀什地区（25 批次）	叶城县（5）、泽普县（10）、疏勒县（10）

监测品种	监测时间	抽样地区及监测批次	
	种10月	**合计：120批次**	
苹果产地土壤	早熟品种7月、中熟品种9月、晚熟品种10月	阿克苏地区（65批次）	红旗坡农场（30）、阿克苏市（20）、温宿县（15）
		伊犁州（30批次）	巩留县（10）、伊宁县（10）、察布查尔县（10）
		喀什地区（25批次）	叶城县（5）、泽普县（10）、疏勒县（10）
		**合计：120批次**	
香梨	9月	巴州（60批次）	库尔勒市（50）、轮台县（5）、尉犁县（5）
		阿克苏地区（30批次）	阿克苏市（10）、库车市（15）、阿瓦提县（5）
		**合计：90批次**	
香梨产地土壤	9月	巴州（60批次）	库尔勒市（50）、轮台县（5）、尉犁县（5）
		阿克苏地区（30批次）	阿克苏市（10）、库车市（15）、阿瓦提县（5）
		**合计：90批次**	
葡萄	吐鲁番地区7月；其他地区8月	吐鲁番市（55批次）	高昌区（25）、鄯善县（30）
		哈密市（5批次）	伊州区（5）
		巴州（5批次）	和硕县（5）
		喀什地区（5批次）	喀什市（5）
		和田地区（10批次）	于田县（5）、墨玉县（5）
		昌吉州（5批次）	昌吉市（5）

监测品种	监测时间	抽样地区及监测批次	
		克州（5 批次）	阿图什市（5）
		合计：90 批次	
葡萄产地土壤	吐鲁番地区 7 月；其他地区 8 月	吐鲁番市（55 批次）	高昌区（25）、鄯善县（30）
		哈密市（5 批次）	伊州区（5）
		巴州（5 批次）	和硕县（5）
		喀什地区（5 批次）	喀什市（5）
		和田地区（10 批次）	于田县（5）、墨玉县（5）
		昌吉州（5 批次）	昌吉市（5）
		克州（5 批次）	阿图什市（5）
		合计：90 批次	
沙棘	9 月	阿勒泰地区（15 批次）	布尔津县（5）、哈巴河县（5）、青河县（5）
		阿克苏地区（15 批次）	温宿县（5）、乌什县（10）
		克州（5 批次）	阿合奇县（5）
		合计：35 批次	
沙棘产地土壤	9 月	阿勒泰地区（15 批次）	布尔津县（5）、哈巴河县（5）、青河县（5）
		阿克苏地区（15 批次）	温宿县（5）、乌什县（10）
		克州（5 批次）	阿合奇县（5）

<table>
<tr><th>监测品种</th><th>监测时间</th><th colspan="2">抽样地区及监测批次</th></tr>
<tr><td></td><td></td><td colspan="2">合计：35 批次</td></tr>
<tr><td rowspan="2">巴旦木</td><td rowspan="2">7 月</td><td>喀什地区（30 批次）</td><td>莎车县（30）</td></tr>
<tr><td colspan="2">合计：30 批次</td></tr>
<tr><td rowspan="2">巴旦木产地土壤</td><td rowspan="2">7 月</td><td>喀什地区（30 批次）</td><td>莎车县（30）</td></tr>
<tr><td colspan="2">合计：30 批次</td></tr>
<tr><td rowspan="5">枸杞</td><td rowspan="5">7 月</td><td>博州（10 批次）</td><td>精河县（10）</td></tr>
<tr><td>塔城地区（2 批次）</td><td>乌苏市（2）</td></tr>
<tr><td>巴州（3 批次）</td><td>尉犁县（3）</td></tr>
<tr><td>昌吉州（5 批次）</td><td>玛纳斯县（5）</td></tr>
<tr><td colspan="2">合计：20 批次</td></tr>
<tr><td rowspan="5">枸杞产地土壤</td><td rowspan="5">7 月</td><td>博州（10 批次）</td><td>精河县（10）</td></tr>
<tr><td>塔城地区（2 批次）</td><td>乌苏市（2）</td></tr>
<tr><td>巴州（3 批次）</td><td>尉犁县（3）</td></tr>
<tr><td>昌吉州（5 批次）</td><td>玛纳斯县（5）</td></tr>
<tr><td colspan="2">合计：20 批次</td></tr>
<tr><td>桃</td><td>7 月</td><td>喀什地区（20 批次）</td><td>疏勒县（10）、英吉沙县（5）、莎车县（5）</td></tr>
</table>

监测品种	监测时间	抽样地区及监测批次	
		和田地区（5批次）	和田县（3）、墨玉县（2）
		**合计：25批次**	
桃产地土壤	7月	喀什地区（20批次）	疏勒县（10）、英吉沙县（5）、莎车县（5）
		和田地区（5批次）	和田县（3）、墨玉县（2）
		**合计：25批次**	
石榴	10月	喀什地区（10批次）	喀什市（5）、伽师县（5）
		和田地区（10批次）	皮山县（5）、策勒县（5）
		**合计：20批次**	
石榴产地土壤	10月	喀什地区（10批次）	喀什市（5）、伽师县（5）
		和田地区（10批次）	皮山县（5）、策勒县（5）
		**合计：20批次**	
新梅	8月	喀什地区（60批次）	伽师县（40）、岳普湖县（10）、莎车县（10）
		伊犁州（10批次）	伊宁县（5）、察布查尔县（5）
		**合计：70批次**	
新梅产地土壤	8月	喀什地区（60批次）	伽师县（40）、岳普湖县（10）、莎车县（10）
		伊犁州（10批次）	伊宁县（5）、察布查尔县（5）

监测品种	监测时间	抽样地区及监测批次	
		**合计：70 批次**	
肉苁蓉	10 月	和田地区（25 批次）	于田县（15）、策勒县（5）、和田县（5）
		巴州（5 批次）	且末县（5）
		**合计：30 批次**	
肉苁蓉产地土壤	10 月	和田地区（25 批次）	于田县（15）、策勒县（5）、和田县（5）
		巴州（5 批次）	且末县（5）
		**合计：30 批次**	
合计		**2200 批次**	

附表 2

抽样数量

产品种类	初检抽样数	复检抽样数
核桃	1000g	1000g
红枣	1000g	1000g
杏	1000g	1000g
苹果	2000g（ >5 颗 ）	2000g（ >5 颗 ）
香梨	1000g	1000g
葡萄	1000g	1000g
沙棘	1000g	1000g
巴旦木	1000g	1000g
枸杞	1000g	1000g
桃	1000g	1000g
石榴	2000g（ >5 颗 ）	2000g（ >5 颗 ）
新梅	1000g	1000g
肉苁蓉	1000g（ >3 根 ）	1000g（ >3 根 ）
土壤	1000g	1000g

备注：抽样数量是指食用林产品去除果皮和果核后的可食用部分需达到的数量。

附表 3

2025 年食用林产品质量安全监测项目及依据标准

产品名称	检测项目	判定依据	检测标准
核桃	农药残留指标（共 26 项）：苯醚甲环唑、二甲戊灵、甲氰菊酯、甲氧滴滴涕、腈苯唑、氯苯甲醚、啶酰菌胺、三氟硝草醚、联苯菊酯、毒死蜱、二嗪磷、伏杀硫磷、三氯杀螨醇、杀虫畏、杀扑磷、速灭磷、肟菌酯、戊唑醇、乙酯杀螨醇、氯氰菊酯和高效氯氰菊酯、氯氟氰菊酯和高效氯氟氰菊酯、亚胺硫磷、乙螨唑、溴氰菊酯、硫丹、氯丹	GB 2763-2021	GB 23200.113
	重金属（共 1 项）：铅	GB 2762-2022	GB 5009.12
红枣	农药残留指标（共 52 项）：艾氏剂、倍硫磷、丙环唑、地虫硫磷、狄氏剂、敌敌畏、对硫磷、伏杀硫磷、咯菌腈、甲胺磷、甲基对硫磷、甲基异柳磷、甲氰菊酯、甲氧滴滴涕、久效磷、抗蚜威、啶酰菌胺、三氟硝草醚、腈菌唑、氧乐果、氯苯甲醚、三氯杀螨醇、杀虫畏、杀扑磷、速灭磷、肟菌酯、乙酯杀螨醇、异狄氏剂、蝇毒磷、治螟磷、氯菊酯、氯唑磷、马拉硫磷、醚菌酯、嘧菌环胺、灭线磷、克百威、乐果、三唑酮、杀螟硫磷、水胺硫磷、硫环磷、氯氰菊酯和高效氯氰菊酯、氟氯氰菊酯和高效氟氯氰菊酯、氰戊菊酯和 S-氰戊菊酯、溴氰菊酯、阿维菌素、啶虫脒、多菌灵、氟酰脲、辛硫磷、苯线磷	GB 2763-2021	GB 23200.121<br>GB 23200.113
	重金属（共 1 项）：铅、镉	GB 2762-2022	GB 5009.12<br>GB 5009.15

产品名称	检测项目	判定依据	检测标准
杏	农药残留指标（共 53 项）：艾氏剂、倍硫磷、地虫硫磷、狄氏剂、敌敌畏、毒死蜱、对硫磷、伏杀硫磷、咯菌腈、甲胺磷、甲基对硫磷、甲基异柳磷、甲氰菊酯、甲氧滴滴涕、腈苯唑、久效磷、抗蚜威、克百威、啶酰菌胺、三氟硝草醚、腈菌唑、氯苯甲醚、三氯杀螨醇、杀虫畏、杀扑磷、速灭磷、肟菌酯、氧乐果、乙酯杀螨醇、异狄氏剂、蝇毒磷、治螟磷、氯菊酯、氯唑磷、马拉硫磷、嘧菌环胺、乐果、嘧霉胺、戊唑醇、杀螟硫磷、水胺硫磷、氯氰菊酯和高效氯氰菊酯、氯氟氰菊酯和高效氯氟氰菊酯、硫环磷、灭线磷、氰戊菊酯和 S-氰戊菊酯、溴氰菊酯、亚胺硫磷、阿维菌素、啶虫脒、氟酰脲、螺螨酯、辛硫磷	GB 2763-2021	GB 23200.121<br>GB 23200.113
	重金属（共 1 项）：铅、镉	GB 2762-2022	GB 5009.12<br>GB 5009.15
苹果	农药残留指标（共 82 项）：百菌清、艾氏剂、倍硫磷、苯醚甲环唑、丙环唑、丙溴磷、哒螨灵、地虫硫磷、狄氏剂、敌敌畏、戊菌唑、毒死蜱、对硫磷、二嗪磷、伏杀硫磷、氟环唑、氟氰戊菊酯、咯菌腈、己唑醇、甲胺磷、甲基对硫磷、甲基异柳磷、甲氰菊酯、甲氧滴滴涕、腈苯唑、久效磷、抗蚜威、啶酰菌胺、三氟硝草醚、腈菌唑、联苯菊酯、氯苯甲醚、三氯杀螨醇、杀虫畏、杀扑磷、速灭磷、肟菌酯、氧乐果、乙酯杀螨醇、异狄氏剂、异菌脲、抑霉唑、蝇毒磷、治螟磷、氯菊酯、氯唑磷、马拉硫磷、醚菌酯、嘧菌环胺、嘧霉胺、灭线磷、克百威、乐果、三唑酮、戊唑醇、乙螨唑、三氯杀螨砜、溴螨酯、三唑醇、烯唑醇、杀螟硫磷、氯苯嘧啶醇、氯氰菊酯和高效氯氰菊酯、氯氟氰菊酯和高效氯氟氰菊酯、氰戊菊酯和 S-氰戊菊酯、硫环磷、三唑磷、水胺硫磷、溴氰菊酯、亚胺硫磷、乙氧氟草醚、阿维菌素、多菌灵、氟酰脲、啶虫脒、甲基硫菌灵、硫线磷、螺螨酯、辛硫磷、苯线磷、吡虫啉、吡唑醚菌酯	GB 2763-2021	NY/T 761<br>GB 23200.121<br>GB 23200.113
	重金属（共 2 项）：铅、镉	GB 2762-2022	GB 5009.12<br>GB 5009.15

产品名称	检测项目	判定依据	检测标准
香梨	农药残留指标（共 77 项）：百菌清、艾氏剂、倍硫磷、地虫硫磷、狄氏剂、敌敌畏、毒死蜱、对硫磷、二嗪磷、伏杀硫磷、氟氰戊菊酯、咯菌腈、己唑醇、甲胺磷、甲基对硫磷、甲基异柳磷、甲氰菊酯、甲氧滴滴涕、腈苯唑、久效磷、抗蚜威、克百威、乐果、联苯菊酯、磷胺、三氟硝草醚、腈菌唑、苯醚甲环唑、戊菌唑、氯苯甲醚、三氯杀螨醇、杀虫畏、杀扑磷、速灭磷、肟菌酯、氧乐果、乙酯杀螨醇、异狄氏剂、异菌脲、抑霉唑、蝇毒磷、治螟磷、氯菊酯、氯唑磷、马拉硫磷、醚菌酯、嘧菌环胺、嘧霉胺、灭线磷、三唑酮、戊唑醇、乙螨唑、溴螨酯、烯唑醇、杀螟硫磷、水胺硫磷、氯氰菊酯和高效氯氰菊酯、溴氰菊酯、氯氟氰菊酯和高效氯氟氰菊酯、氟氯氰菊酯和高效氟氯氰菊酯、硫环磷、氯苯嘧啶醇、氰戊菊酯和 S-氰戊菊酯、亚胺硫磷、阿维菌素、甲基硫菌灵、多菌灵、氟酰脲、啶虫脒、硫线磷、螺螨酯、辛硫磷、唑螨酯、苯线磷、吡虫啉、吡唑醚菌酯、除虫脲	GB 2763-2021	NY/T 761<br>GB 23200.121<br>GB 23200.113
	重金属（共 1 项）：铅、镉	GB 2762-2022	GB 5009.12<br>GB 5009.15
葡萄	农药残留指标（共 76 项）：百菌清、艾氏剂、倍硫磷、苯醚甲环唑、地虫硫磷、狄氏剂、敌敌畏、毒死蜱、对硫磷、氟环唑、腐霉利、咯菌腈、己唑醇、甲胺磷、甲基对硫磷、甲基异柳磷、甲氰菊酯、甲氧滴滴涕、腈苯唑、久效磷、抗蚜威、克百威、喹氧灵、乐果、联苯菊酯、磷胺、啶酰菌胺、三氟硝草醚、腈菌唑、氯苯甲醚、氯菊酯、氯唑磷、马拉硫磷、醚菌酯、嘧菌环胺、嘧霉胺、灭线磷、三氯杀螨醇、三唑酮、杀虫畏、速灭磷、肟菌酯、戊菌唑、氧乐果、乙酯杀螨醇、异狄氏剂、异菌脲、抑霉唑、蝇毒磷、治螟磷、三唑醇、杀螟硫磷、水胺硫磷、氯苯嘧啶醇、戊唑醇、乙螨唑、烯唑醇、溴螨酯、氯氰菊酯和高效氯氰菊酯、氯氟氰菊酯和高效氯氟氰菊酯、氰戊菊酯和 S-氰戊菊酯、溴氰菊酯、亚胺硫磷、阿维菌素、啶虫脒、多菌灵、甲基硫菌灵、硫线磷、螺螨酯、内吸磷、辛硫磷、唑螨酯、苯线磷、吡虫啉、吡唑醚菌酯、庚烯磷	GB 2763-2021	NY/T 761<br>GB 23200.121<br>GB 23200.113

产品名称	检测项目	判定依据	检测标准
	重金属（共 1 项）：铅、镉	GB 2762-2022	GB 5009.12<br>GB 5009.15
沙棘	农药残留指标（共 43 项）：倍硫磷、敌敌畏、地虫硫磷、对硫磷、甲胺磷、甲基对硫磷、甲基异柳磷、甲氰菊酯、甲氧滴滴涕、抗蚜威、克百威、乐果、磷胺、硫环磷、氯苯甲醚、氯菊酯、氯唑磷、嘧菌环胺、嘧霉胺、灭线磷、三氯杀螨醇、啶酰菌胺、三氟硝草醚、杀虫畏、杀扑磷、速灭磷、氧乐果、乙酯杀螨醇、蝇毒磷、治螟磷、艾氏剂、狄氏剂、杀螟硫磷、氯氟氰菊酯和高效氯氟氰菊酯、久效磷、氰戊菊酯和 S-氰戊菊酯、水胺硫磷、异狄氏剂、啶虫脒、多菌灵、硫线磷、吡虫啉、辛硫磷	GB 2763-2021	GB 23200.121<br>GB 23200.113
	重金属（共 1 项）：铅、镉	GB 2762-2022	GB 5009.12<br>GB 5009.15
巴旦木	农药残留指标（共 22 项）：苯醚甲环唑、二甲戊灵、甲氰菊酯、啶酰菌胺、三氟硝草醚、三氯杀螨醇、杀虫畏、杀扑磷、速灭磷、肟菌酯、甲氧滴滴涕、腈苯唑、氯苯甲醚、联苯菊酯、戊唑醇、氯氰菊酯和高效氯氰菊酯、氯氟氰菊酯和高效氯氟氰菊酯、硫丹、亚胺硫磷、乙螨唑、乙酯杀螨醇、氯丹	GB 2763-2021	GB 23200.113
	重金属（共 1 项）：铅	GB 2762-2022	GB 5009.12
枸杞	农药残留指标（共 48 项）：百菌清、倍硫磷、敌敌畏、地虫硫磷、对硫磷、甲胺磷、甲基对硫磷、甲基异柳磷、甲氰菊酯、甲氧滴滴涕、抗蚜威、克百威、乐果、磷胺、硫环磷、氯苯甲醚、氯菊酯、氯唑磷、嘧菌环胺、嘧霉胺、灭线磷、三氯杀螨醇、啶酰菌胺、三氟硝草醚、杀虫畏、速灭磷、氧乐果、乙酯杀螨醇、蝇毒磷、治螟磷、艾氏剂、狄氏剂、异狄氏剂、哒螨灵、毒死蜱、己唑醇、乙螨唑、氯氟氰菊酯和高效氯氟氰菊酯、久效磷、氰戊菊酯和 S-氰戊菊酯、杀螟硫磷、水胺硫磷、阿维菌素、啶虫脒、多菌灵、硫	GB 2763-2021	NY/T 761<br>GB 23200.121<br>GB 23200.113

产品名称	检测项目	判定依据	检测标准
	线磷、辛硫磷、唑螨酯		
	重金属（共1项）：铅、镉	GB 2762-2022	GB 5009.12<br>GB 5009.15
桃	农药残留指标（共63项）：百菌清、艾氏剂、倍硫磷、苯醚甲环唑、丙环唑、地虫硫磷、狄氏剂、敌敌畏、毒死蜱、对硫磷、二嗪磷、伏杀硫磷、咯菌腈、甲胺磷、甲基对硫磷、甲基异柳磷、甲氰菊酯、甲氧滴滴涕、腈苯唑、久效磷、啶酰菌胺、三氟硝草醚、腈菌唑、抗蚜威、克百威、乐果、氯苯甲醚、氯菊酯、氯唑磷、马拉硫磷、嘧菌环胺、嘧霉胺、灭线磷、三氯杀螨醇、杀虫畏、杀扑磷、速灭磷、肟菌酯、戊菌唑、氧乐果、乙酯杀螨醇、异狄氏剂、异菌脲、蝇毒磷、治螟磷、杀螟硫磷、水胺硫磷、戊唑醇、硫环磷、氯苯嘧啶醇、氯氰菊酯和高效氯氰菊酯、氯氟氰菊酯和高效氯氟氰菊酯、氟氯氰菊酯和高效氟氯氰菊酯、氰戊菊酯和S-氰戊菊酯、溴氰菊酯、亚胺硫磷、阿维菌素、啶虫脒、多菌灵、氟酰脲、螺螨酯、辛硫磷、苯线磷	GB 2763-2021	NY/T 761<br>GB 23200.121<br>GB 23200.113
	重金属（共1项）：铅、镉	GB 2762-2022	GB 5009.12<br>GB 5009.15
石榴	农药残留指标（共40项）：倍硫磷、敌敌畏、地虫硫磷、对硫磷、甲胺磷、甲基对硫磷、甲基异柳磷、甲氰菊酯、甲氧滴滴涕、久效磷、克百威、乐果、磷胺、硫环磷、氯苯甲醚、氯菊酯、氯唑磷、灭线磷、三氯杀螨醇、杀虫畏、杀螟硫磷、杀扑磷、水胺硫磷、速灭磷、三氟硝草醚、氧乐果、乙酯杀螨醇、蝇毒磷、治螟磷、艾氏剂、异狄氏剂、苯醚甲环唑、咯菌腈、氰戊菊酯和S-氰戊菊酯、狄氏剂、啶虫脒、硫线磷、吡虫啉、内吸磷、辛硫磷	GB 2763-2021	GB 23200.121<br>GB 23200.113
	重金属（共1项）：铅、镉	GB 2762-2022	GB 5009.12<br>GB 5009.15

产品名称	检测项目	判定依据	检测标准
新梅	农药残留指标（共 46 项）：艾氏剂、倍硫磷、地虫硫磷、狄氏剂、敌敌畏、对硫磷、伏杀硫磷、咯菌腈、甲胺磷、甲基对硫磷、甲基异柳磷、甲氰菊酯、甲氧滴滴涕、久效磷、克百威、乐果、硫环磷、啶酰菌胺、三氟硝草醚、腈菌唑、氯苯甲醚、氯菊酯、氯唑磷、嘧菌环胺、灭线磷、三氯杀螨醇、杀虫畏、杀扑磷、速灭磷、肟菌酯、氧乐果、杀螟硫磷、乙酯杀螨醇、水胺硫磷、蝇毒磷、氯氰菊酯和高效氯氰菊酯、氰戊菊酯和S-氰戊菊酯、异狄氏剂、治螟磷、啶虫脒、氟酰脲、辛硫磷、内吸磷、唑螨酯、苯线磷、庚烯磷	GB 2763-2021	GB 23200.121<br>GB 23200.113
	重金属（共 1 项）：铅、镉	GB 2762-2022	GB 5009.12<br>GB 5009.15
肉苁蓉	农药残留指标（共 13 项）：甲基异柳磷、甲氧滴滴涕、克百威、乐果、氯苯甲醚、三氯杀螨醇、杀虫畏、杀扑磷、乙酯杀螨醇、三氟硝草醚、速灭磷、庚烯磷、氯磺隆	GB 2763-2021	GB 23200.121<br>GB 23200.113

附表 4

2025 年产地土壤质量安全监测项目及依据标准

产品名称	序号	检测项目	风险筛选值（≤mg/kg）				依据方法	检测方法标准
			pH≤5.5	5.5＜pH≤6.5	6.5＜pH≤7.5	pH＞7.5		
产地土壤	1	镉	0.3	0.3	0.3	0.6	GB 15618-2018	GB/T 17141
	2	汞	1.3	1.8	2.4	3.4		NY/T 1121.10
	3	砷	40	40	30	25		NY/T 1121.11
	4	铅	70	90	120	170		GB/T 17141
	5	铬	150	150	200	250		HJ 491
	6	铜	150	150	200	200		HJ 491
	7	镍	60	70	100	190		HJ 491
	8	锌	200	200	250	250		HJ 491
	9	六六六总量	0.10					GB/T 14550
	10	滴滴涕总量	0.10					GB/T 14550
	11	苯并[a]芘	0.55					HJ 805

注：①六六六总量为α-六六六、β-六六六、γ-六六六、δ-六六六四种异构体的含量总和。
②滴滴涕总量为 p,p'-滴滴伊、p,p'-滴滴滴、o,p'-滴滴涕、p,p'-滴滴涕四种衍生物的含量总和。
